# Network-level reprogramming of cell death pathways in colorectal cancer cells by combined thymoquinone and 5-fluorouracil treatment

**DOI:** 10.3389/fmolb.2026.1864680

**Published:** 2026-06-24

**Authors:** Natalia Kurowska, Celina Kruszniewska-Rajs, Barbara Strzałka-Mrozik

**Affiliations:** Department of Molecular Biology, Faculty of Pharmaceutical Sciences in Sosnowiec, Medical University of Silesia, Katowice, Poland

**Keywords:** 5-fluorouracil, cell death pathways, colorectal cancer, drug combination, signaling networks, stress response, thymoquinone, transcriptomic profiling

## Abstract

**Introduction:**

Colorectal cancer remains a leading cause of cancer-related mortality, with resistance to 5-fluorouracil (5-FU) posing a major therapeutic challenge. Thymoquinone (TQ), a bioactive compound derived from *Nigella sativa*, exhibits anticancer activity; however, its system-level effects in colorectal cancer are not fully understood.

**Methods:**

RKO colorectal cancer cells were treated with TQ, 5-FU, or their combination for 24 h, followed by genome-wide transcriptomic profiling using oligonucleotide microarrays. Drug interaction effects were assessed using a deviation-from-additivity model. Selected genes were validated by RT-qPCR and Western blotting, and functional relevance was evaluated using bioinformatics analyses.

**Results:**

Combined treatment induced extensive network-level reprogramming of pathways associated with apoptosis, cellular stress response, and proliferation. Although no classical transcriptional synergy was observed, the interaction between TQ and 5-FU resulted in coordinated modulation of overlapping signaling networks. Key regulatory genes, including *FAS* and *CYLD*, were linked to enhanced pro-apoptotic signaling, whereas *BIRC3* and *EIF2AK3* were associated with adaptive or resistance-related responses.

**Conclusion:**

TQ acts as a context-dependent modulator of chemotherapy response, reshaping cell death and stress-related signaling networks rather than directly enhancing cytotoxicity. These findings highlight the potential of TQ to influence therapeutic responses in fluoropyrimidine-based treatment of colorectal cancer and support further functional and *in vivo* validation.

## 1 Introduction

Colorectal cancer (CRC) remains a leading cause of cancer-related mortality worldwide, accounting for nearly 10% of all cancer incidence and deaths. Despite advances in screening and treatment, the incidence of CRC particularly among younger individuals continues to rise, highlighting the need for improved therapeutic strategies (Siegel et al., 2026; Sinicrope, 2022). Most CRC cases are associated with modifiable risk factors, including diet, obesity, and lifestyle, whereas a smaller proportion arises from inherited genetic mutations (Kanth and Inadomi, 2021). Approximately 6%–10% of CRC cases are attributable to known germline mutations (Kanth and Inadomi, 2021).

At the molecular level, CRC is a heterogeneous disease classified into four consensus molecular subtypes (CMS1–4), each characterized by distinct biological features and therapeutic responses (Guinney et al., 2015; Kantha et al., 2025). This heterogeneity contributes to variable treatment outcomes and represents a major challenge in the development of effective therapeutic approaches.

Fluoropyrimidine-based chemotherapy, particularly 5-fluorouracil (5-FU), remains the cornerstone of systemic CRC treatment. However, its clinical efficacy is limited by intrinsic and acquired resistance, with only a subset of patients achieving durable responses (Chauvin et al., 2022; Morris et al., 2023). Moreover, resistance to 5-FU is frequently associated with cross-resistance to other cytotoxic agents, underscoring the need for novel strategies that can modulate treatment response rather than relying solely on increased cytotoxicity.

Given the limitations of conventional chemotherapy, increasing attention has been directed toward novel therapeutic approaches aimed not only at directly eliminating tumor cells but also at reprogramming dysregulated cellular pathways and modulating the tumor microenvironment (TME). In colorectal cancer, emerging evidence suggests that simultaneous targeting of molecular signaling networks and immune components of the TME may enhance therapeutic efficacy and help overcome treatment resistance. Recent studies indicate that modulation of tumor-associated macrophage (TAM) polarization and promotion of CD8^+^ T-cell infiltration can improve anti-tumor immunity and support the development of innovative combination-based therapeutic strategies in CRC (Wang et al., 2025).

Natural compounds have gained increasing attention as modulators of cancer-related signaling pathways. Thymoquinone (TQ), the principal bioactive component of *Nigella sativa*, exhibits a broad spectrum of biological activities in both *in vitro* and *in vivo* models, including antioxidant, antimicrobial, anti-inflammatory, hepatoprotective, immunomodulatory, and neuroprotective effects (Alam et al., 2023; Ali Bakr and Saad Alyamani, 2023; Barashkova et al., 2023; Ciesielska-Figlon et al., 2023; Gholamnezhad et al., 2016). In addition, TQ has demonstrated antiproliferative and pro-apoptotic activity across multiple cancer types, including colorectal cancer (Al-Rawashde et al., 2023; Liu et al., 2017; Nithya et al., 2023; Rajput et al., 2013; Şahin et al., 2023; Kurowska et al., 2023; Al Bitar et al., 2025; Ballout et al., 2020; Zeinali et al., 2024).

Its pleiotropic mode of action and relatively low toxicity make TQ an attractive candidate for combination therapy. However, despite growing evidence supporting its anticancer activity, the system-level effects of TQ particularly in combination with 5-FU remain insufficiently characterized.

The RKO colorectal cancer cell line represents a distinct molecular subtype characterized by microsatellite instability, the BRAF V600E mutation, and wild-type APC, β-catenin, and TP53, as well as a high mutation burden, making it a relevant model for studying stress response and cell death–related signaling (Madej et al., 2025). While previous studies have demonstrated the cytotoxic effects of thymoquinone in colorectal cancer models, comprehensive transcriptomic analyses of its combinatorial effects with 5-fluorouracil in this context remain limited. In particular, it is unclear whether thymoquinone enhances the cytotoxic effects of 5-fluorouracil through classical synergistic mechanisms or rather modulates the cellular response at the level of signaling networks. These limitations underscore the need for system-level approaches to elucidate how TQ modulates chemotherapy response at the level of signaling networks.

In this context, we hypothesized that thymoquinone modulates the transcriptional response to 5-fluorouracil by reprogramming signaling networks associated with cell death and stress response, with particular emphasis on TNF-centered regulatory pathways governing the balance between apoptosis, survival, and adaptive stress signaling. Importantly, this framework assumes that the combined effect of both agents is not necessarily reflected by increased cytotoxicity or transcriptional synergy, but rather by coordinated network-level modulation of overlapping signaling pathways.

To address this, we performed genome-wide transcriptomic profiling of colorectal cancer cells treated with thymoquinone, 5-fluorouracil, and their combination, followed by integrative bioinformatics and experimental validation. This approach was designed to determine whether thymoquinone functions as a context-dependent modulator of chemotherapy response through transcriptional rewiring of cell fate–related signaling networks.

## 2 Materials and methods

### 2.1 Cell culture conditions

The human colorectal adenocarcinoma cell line RKO (ATCC® CRL-2577) was cultured in Eagle’s Minimum Essential Medium (EMEM; Sigma-Aldrich, Merck, St. Louis, MO, United States) supplemented with 10% fetal bovine serum (Euroclone SpA, Pero, Italy) and gentamicin (50 μg/mL; Sigma-Aldrich, Merck, St. Louis, MO, United States). Cells were maintained at 37 °C in a humidified atmosphere containing 5% CO_2_ in a CO_2_ incubator (Thermo Fisher Scientific, Waltham, MA, United States) (Capes-Davis et al., 2021).

The culture medium was replaced every 2–3 days, and cells were subcultured upon reaching approximately 80% confluence using 0.25% trypsin–EDTA (Sigma-Aldrich, Merck, St. Louis, MO, United States). Experiments were performed using cells within six passages to ensure phenotypic stability and reproducibility.

Cell morphology and viability were routinely monitored, and all cultures were confirmed to be free of *mycoplasma* contamination using a PCR-based assay before experimental procedures.

### 2.2 Compound preparation and cell treatment

Cells were seeded in six-well culture plates (Thermo Fisher Scientific, Waltham, MA, United States) at a density of 3 × 10^5^ cells per well and incubated for 48 h to allow attachment and stabilization. Cells were subsequently treated with thymoquinone, 5-fluorouracil, or their combination.

Thymoquinone (Sigma-Aldrich, St. Louis, MO, United States) was applied at a final concentration of 20 μM, while 5-fluorouracil (Sigma-Aldrich, St. Louis, MO, United States) was used at 10 μg/mL (approximately 76.8 µM). These concentrations were selected based on previously published dose–response, cell viability, and apoptosis analyses performed in RKO colorectal cancer cells under comparable experimental conditions (Kurowska et al., 2026). In that study, thymoquinone exhibited a concentration-dependent cytotoxic effect, with an estimated IC_50_ value of approximately 37 µM in RKO cells. In contrast, IC_50_ values were not determined for 5-FU or the combination treatment because cell viability did not decrease below 50% within the tested concentration range. A reduction in cell viability below 70% was considered indicative of a biologically relevant cytotoxic response. The concentrations used in the present study were selected to induce measurable biological responses while maintaining sufficient cell viability for transcriptomic profiling. The rationale for concentration selection and the corresponding biological response data are provided in Supplementary Table S1.

In the combination group, both compounds were administered simultaneously at the indicated concentrations. The concentrations used in this study were selected to induce measurable transcriptional responses under controlled *in vitro* conditions and should not be interpreted as direct equivalents of clinically achievable plasma concentrations, particularly in the case of thymoquinone.

Stock solutions were prepared in dimethyl sulfoxide (DMSO; Sigma-Aldrich, St. Louis, MO, United States), and the final DMSO concentration in all experimental conditions did not exceed 1% (v/v). Control cells were treated with an equivalent concentration of DMSO. Prior to use, working solutions were sterilized using 0.2 µm syringe filters (Sartorius, Göttingen, Germany). Following 24 h of treatment, cells were harvested for subsequent transcriptomic analysis.

### 2.3 Microarray-based transcriptomic profiling

Cells treated with TQ, 5-FU, or their combination were subjected to genome-wide gene expression analysis. Total RNA was isolated from RKO cells using TRIzol reagent (Invitrogen Life Technologies, Carlsbad, CA, United States) according to the manufacturer’s protocol. RNA concentration and purity were assessed using a MaestroNano MN-913 spectrophotometer (MaestroGen Inc., Las Vegas, NV, United States), whereas RNA integrity was additionally evaluated by capillary gel electrophoresis. A total of 250 ng RNA was used per sample for microarray analysis.

Transcriptomic profiling was performed using the Affymetrix Human Genome U133A 2.0 Array (Affymetrix, Santa Clara, CA, United States) according to the manufacturer’s GeneChip expression analysis protocol. Total RNA was processed using the GeneChip™ 3′ IVT PLUS Reagent Kit (Thermo Fisher Scientific, Waltham, MA, United States), recommended for use with the HG-U133A 2.0 platform, to generate amplified and biotin-labeled complementary RNA (cRNA).

Total RNA was reverse-transcribed into single-stranded cDNA using a T7 oligo (dT) primer. The resulting single-stranded cDNA was subsequently converted into double-stranded cDNA, which served as a template for T7-based *in vitro* transcription (IVT). This process generated biotin-labeled cRNA through linear amplification. The resulting cRNA was fragmented and hybridized to oligonucleotide probes immobilized on the Affymetrix microarray.

Following hybridization, arrays were stained with streptavidin–phycoerythrin using the GeneChip™ Fluidics Station 450 (Thermo Fisher Scientific, Waltham, MA, United States) and scanned with the GeneChip® Scanner 3000 (Affymetrix, Santa Clara, CA, United States) to obtain fluorescence signal intensities.

Raw microarray data were processed and analyzed using GeneSpring GX 13.0 software (Agilent Technologies United Kingdom Limited, South Queensferry, United Kingdom) within the PLGrid Infrastructure (https://www.plgrid.pl/). Data preprocessing included background correction, normalization, and summarization using the Robust Multi-array Average (RMA) algorithm. Differential gene expression analysis was performed by comparing treated samples with untreated controls. Genes were considered differentially expressed at a fold change (FC) ≥ 2 and a false discovery rate (FDR)-adjusted p-value <0.05.

### 2.4 Analysis of drug interaction effects

To estimate the interaction effect between TQ and 5-FU, a deviation-from-additivity approach was applied using log_2_ fold change (logFC) values derived from microarray analyses. For each gene, the interaction term was calculated according to the formula:

$$Interaction={logFC}_{TQ+5-FU}-{logFC}_{TQ}-{logFC}_{5-FU}$$

where logFC_TQ+5-FU_, logFC_TQ_, and logFC_5-FU_ represent gene expression changes relative to untreated control cells.

This approach enables estimation of the extent to which the transcriptional response to the combined treatment deviates from the expected additive effect of the individual treatments. Positive interaction values indicate responses exceeding additive expectations, whereas negative values reflect responses below the expected additive level (Chou, 2010; Geva-Zatorsky et al., 2010). The additive expectation was used as a reference (null) model for identifying deviations in transcriptional responses and was not intended as a measure of classical pharmacological synergy. Unlike models such as Loewe additivity or Bliss independence, which are typically applied to dose–response or phenotypic endpoints (e.g., cell viability or growth inhibition), the present approach was designed to evaluate interactions at the transcriptomic level across thousands of genes.

Importantly, the additive model does not assume shared biological mechanisms between TQ and 5-FU, but rather provides a reference framework for assessing whether the combined transcriptional response differs from that expected under independent additive effects.

### 2.5 RT-qPCR validation of microarray results

To validate gene expression changes identified in the microarray analysis, real-time reverse transcription quantitative PCR (RT-qPCR) was performed for selected genes. Genes were selected based on a fold change greater than 2 (FC > 2) in the microarray dataset and their involvement in key pathways related to cell death regulation.

Relative mRNA expression levels were calculated using the 2^−ΔCT^ method. Ct values for target genes were normalized to the geometric mean of the reference genes *GAPDH* and *TBP* to obtain ΔCt values (Livak and Schmittgen, 2001). Amplification of target and reference genes was performed in parallel to ensure accurate comparative quantification.

RT-qPCR reactions were carried out using the SensiFAST™ SYBR® Hi-ROX One-Step Kit (Bioline, London, United Kingdom), based on SYBR Green dye chemistry, which enables real-time detection of PCR product accumulation through fluorescence binding to double-stranded DNA. Primer sequences used in this study are listed in Table 1.

**TABLE 1:** Primer sequences, expected amplicon sizes, and gene accession numbers used for RT-qPCR analysis.

Gene	Forward primer (5′→ 3′)	Reverse primer (5′→ 3′)	Gene accession number	Product length (bp)
*CYLD*	GGTAATCCGTTGGATCGGTCAG	AGTGCCTCTGAAGGTTCCATCC	NM_015247	106
*BIRC3*	TTTCCGTGGCTCTTATTCAAACT	GCACAGTGGTAGGAACTTCTCAT	NM_001165.5	96
*STK3*	GGCAGATTTTGGAGTGGCTGGT	AATGCCAAGGGACCAGATGTCG	NM_006281.4	142
*EIF2AK3*	ACGATGAGACAGAGTTGCGAC	ATCCAAGGCAGCAATTCTCCC	NM_004836.7	80
*ID2*	TTGTCAGCCTGCATCACCAGAG	AGCCACACAGTGCTTTGCTGTC	NM_002166.5	150
*GAPDH*	GAAGGTGAAGGTCGGAGTC	GAAGATGGTGATGGGATTTC	NM_002046.7	226
*TBP*	GCCAAGAGTGAAGAACAG	GAAGTCCAAGAACTTAGCTG	NM_003194.5	90

Amplification was performed using a LightCycler® 480 System (Roche, Basel, Switzerland) according to the manufacturer’s instructions. The thermal cycling protocol included a reverse transcription step at 45 °C for 10 min, followed by initial denaturation at 95 °C for 2 min and 45 amplification cycles. Each cycle consisted of denaturation at 95 °C for 5 s, annealing at 60 °C for 10 s, and extension at 72 °C for 5 s.

A melting curve analysis was performed at the end of each run to verify amplification specificity. RT-qPCR experiments were conducted using three independent biological replicates, each analyzed in triplicate as technical replicates, in accordance with accepted methodological guidelines (Rieu and Powers, 2009).

### 2.6 Western blot analysis of protein expression

Cells were lysed in RIPA buffer (Sigma-Aldrich, Merck, St. Louis, MO, United States) supplemented with protease and phosphatase inhibitor cocktails. Lysates were incubated on ice for 30 min, centrifuged, and the supernatants were collected. Protein concentrations were determined using a BCA Protein Assay Kit (Sigma-Aldrich, Merck, St. Louis, MO, United States).

A total of 5 μg of protein per sample was mixed with Laemmli sample buffer containing β-mercaptoethanol, denatured at 95 °C for 5 min, and separated by SDS–PAGE using 4% stacking and 10% resolving gels. Proteins were then transferred onto nitrocellulose membranes (Bio-Rad Laboratories, Inc., Hercules, CA, United States) using a semi-dry transfer system.

Membranes were blocked with 5% non-fat milk in TBST (TBS containing 0.1% Tween-20) for 30 min at room temperature and incubated overnight at 4 °C with primary antibodies against Fas (1:500; Invitrogen, Waltham, MA, United States; catalog no. MA5-14882) and β-actin (1:5000; Invitrogen, Waltham, MA, United States; catalog no. MA1-91399), which served as the loading control. After washing, membranes were incubated for 1.5 h at room temperature with HRP-conjugated secondary antibodies: anti-mouse IgG (1:500; Sigma-Aldrich, catalog no. A4416) and anti-rabbit IgG (1:2000; Cell Signaling Technology, Danvers, MA, United States; catalog no. 7074S) (Begum et al., 2022).

Protein bands were detected using an enhanced chemiluminescence substrate (Pierce™ ECL, Thermo Fisher Scientific, Waltham, MA, United States) and visualized with an iBright FL1500 imaging system (Thermo Fisher Scientific, Waltham, MA, United States). Band intensities were normalized to β-actin and quantified using densitometric analysis. Experiments were performed in three independent biological replicates.

### 2.7 *In silico* functional enrichment and network analysis

To further explore the biological significance of the identified genes, a series of *in silico* bioinformatics analyses were performed. Functional enrichment analysis was conducted using the g:Profiler platform (https://biit.cs.ut.ee/gprofiler) to identify significantly overrepresented Gene Ontology (GO) biological processes and Reactome pathways. Statistical significance was determined using the built-in g:SCS multiple testing correction with a threshold of p < 0.05.

Protein–protein interaction (PPI) networks were constructed using the STRING database (version 12.0; https://string-db.org) to evaluate functional relationships among differentially expressed genes. Networks were generated using a confidence score threshold of 0.4 (medium confidence), and both direct (physical) and indirect (functional) associations were included.

Additional gene interaction networks were generated using GeneMANIA (http://genemania.org) to further investigate functional relationships, including co-expression, shared protein domains, pathway co-membership, and genetic interactions.

To assess the clinical relevance of selected candidate genes, their expression levels in colorectal cancer tissues were analyzed using the UALCAN platform (http://ualcan.path.uab.edu), which provides access to gene expression data derived from The Cancer Genome Atlas (TCGA) project. Comparative analyses between tumor and normal tissue samples were performed using default parameters, including Student’s t-test for statistical comparisons.

### 2.8 Statistical analysis

Statistical analyses were performed using STATISTICA software (version 13.3; TIBCO Software Inc., Palo Alto, CA, United States). A p-value <0.05 was considered statistically significant. Data normality was assessed using the Shapiro–Wilk test.

RT-qPCR data were analyzed using the non-parametric Kruskal–Wallis test followed by *post hoc* pairwise comparisons, due to the non-normal distribution of the data (Conover, 1999). Results are presented as medians with interquartile range (IQR).

Western blot densitometric data were analyzed using one-way analysis of variance (ANOVA) followed by Tukey’s *post hoc* test for multiple comparisons.

Differential gene expression analysis of microarray data was performed using the statistical framework implemented in GeneSpring GX 13.0 (Agilent Technologies United Kingdom Limited, South Queensferry, United Kingdom). Expression values were analyzed at the individual transcript (probe set) level. GeneSpring first applied one-way analysis of variance (ANOVA) across all experimental groups, followed by Tukey’s honestly significant difference (HSD) *post hoc* test to identify significant pairwise differences between groups. P-values were adjusted for multiple testing using the Benjamini–Hochberg false discovery rate (FDR) correction.

For volcano plot generation, pairwise comparisons between each treatment group and the control group were additionally performed using Student’s t-test. The resulting p-values and fold change values were used for volcano plot visualization.

For downstream functional analyses, including pathway enrichment and candidate gene selection, an additional fold change threshold of FC ≥ 2 was applied. Thus, transcripts were selected for downstream analyses when they met both the significance criterion (p < 0.05) and the fold change threshold (FC ≥ 2).

The sample size used in this study was consistent with commonly applied standards in transcriptomic analyses. Although no formal power calculation was performed, the inclusion of independent biological replicates and validation using complementary experimental approaches was intended to ensure the reliability of the results.

## 3 Results

### 3.1 Identification of differentially expressed genes

Genome-wide gene expression profiling was performed to assess transcriptional changes in RKO colorectal cancer cells following treatment with TQ, 5-FU, and their combination. A total of 22,277 transcripts were analyzed.

**FIGURE 1:**
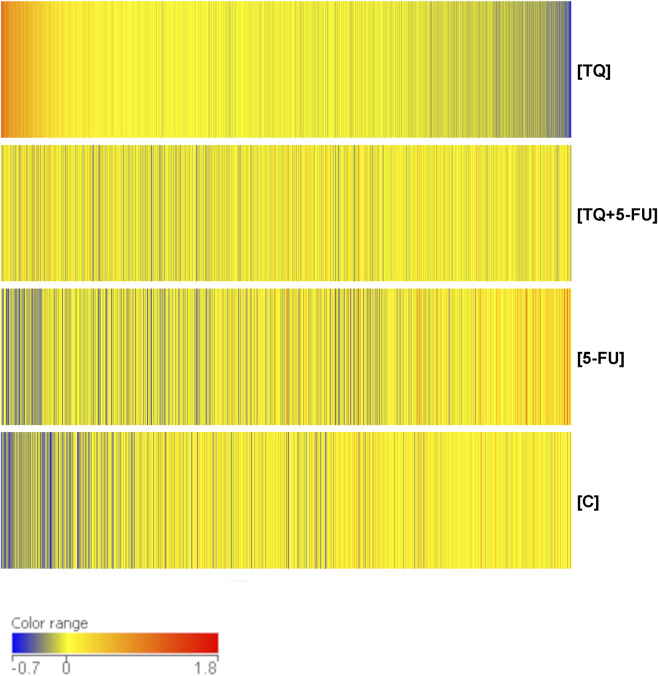
Heatmap of global gene expression profiles in RKO colorectal cancer cells following treatment with thymoquinone, 5-fluorouracil, and their combination. Hierarchical clustering reveals distinct transcriptional patterns across experimental groups, indicating treatment-specific modulation of gene expression. [C] – control (untreated cells); [TQ] – thymoquinone; [5-FU] – 5-fluorouracil; [TQ+5-FU] – combined treatment.

Hierarchical clustering and heatmap visualization revealed distinct gene expression patterns across experimental groups, indicating substantial transcriptional reprogramming in response to treatment (Figure 1). Differential gene expression was determined by comparing normalized expression values between treated and control cells. Hierarchical clustering demonstrated clear separation between treatment groups and control samples, supporting treatment-specific transcriptional responses.

Significant differences in transcript expression were identified among the experimental groups using one-way ANOVA followed by Tukey’s HSD *post hoc* analysis (Table 2). Of the 22,277 transcripts analyzed, 7,315 exhibited significant differences in expression across the experimental groups according to the overall ANOVA analysis.

**TABLE 2:** Differentially expressed transcripts identified between experimental groups using one-way ANOVA followed by Tukey’s HSD *post hoc* test. Comparisons were performed between control (CON), thymoquinone-treated (TQ), 5-fluorouracil-treated (5-FU), and combination-treated (TQ+5-FU) cells. Gray cells indicate the total number of transcripts identified as significantly different across all experimental groups by one-way ANOVA (n = 7,315). Blue cells indicate transcripts that were significantly differentially expressed (p < 0.05) in Tukey’s HSD *post hoc* comparisons between the group indicated in the row and the group indicated in the column; numbers in parentheses represent downregulated (↓) and upregulated (↑) transcripts. Red cells indicate transcripts from the ANOVA-significant set that did not differ significantly in the corresponding pairwise comparison.

Group name	TQ	TQ+5-FU	5-FU	CON
TQ	7,315	2,684 (1,873 ↓ 811 ↑)	4,704 (2,503 ↓ 2,201 ↑)	4,061 (2,259 ↓ 1,802 ↑)
TQ+5-FU	4,631	7,315	2,418 (1,169 ↓ 1,249 ↑)	4,705 (2,402 ↓ 2,303 ↑)
5-FU	2,611	4,897	7,315	4,093 (2,141 ↓ 1,952 ↑)
CON	3,254	3,222	2,610	7,315

The matrix is read as row-versus-column comparisons. The upper triangle summarizes significantly differentially expressed transcripts identified by Tukey’s HSD, *post hoc* test, whereas the lower triangle shows transcripts that were not significantly differentially expressed in the corresponding pairwise comparisons.

A substantial number of transcripts were significantly altered in each treatment group compared with untreated controls. Specifically, 4,061 transcripts were differentially expressed following thymoquinone treatment, 4,705 transcripts following 5-fluorouracil treatment, and 4,093 transcripts in the combined treatment group (TQ+5-FU).

Notably, 5-FU treatment induced the highest number of transcriptional changes, whereas the combination treatment resulted in a comparable but not increased number of differentially expressed genes, suggesting overlapping or convergent transcriptional responses rather than additive effects.

To identify transcripts with both statistical and biological relevance, subsequent pairwise comparisons were filtered using a fold change threshold of FC ≥ 2.0 in addition to statistical significance (p < 0.05). Only transcripts meeting both criteria were included in downstream analyses, including functional characterization and candidate selection for RT-qPCR validation.

### 3.2 Thymoquinone

**FIGURE 2:**
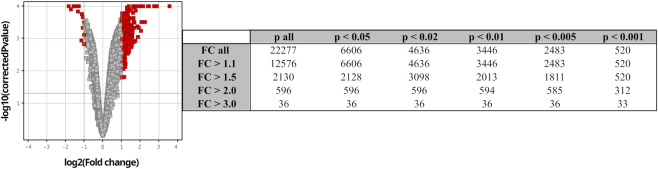
Volcano plot of differential gene expression in RKO colorectal cancer cells following thymoquinone treatment compared with control cells. Differentially expressed transcripts are highlighted in red. The accompanying table summarizes the number of transcripts meeting defined fold change (FC) and statistical significance thresholds.

Transcriptomic analysis revealed substantial changes in transcript expression following thymoquinone treatment compared with control cells. A total of 596 transcripts were significantly differentially expressed at a fold change threshold of FC ≥ 2.0 (p < 0.05), including 36 transcripts with pronounced expression changes exceeding FC ≥ 3.0 (Figure 2). The number of differentially expressed transcripts decreased with increasing fold change thresholds, reflecting the identification of a core subset of strongly regulated transcripts.

**FIGURE 3:**
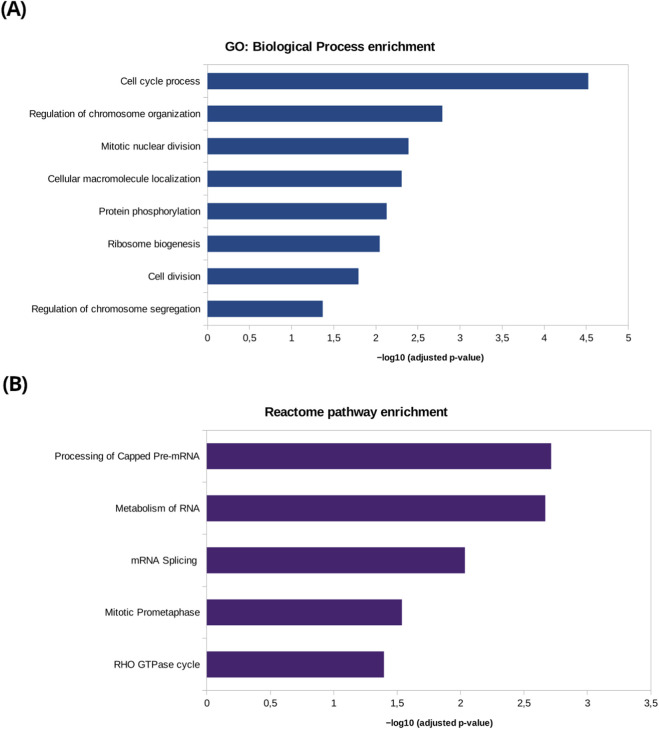
Functional enrichment analysis of transcripts differentially expressed in RKO colorectal cancer cells following thymoquinone treatment. **(A)** Significantly enriched GO Biological Process categories. **(B)** Significantly enriched Reactome pathways, highlighting processes related to cell cycle regulation and RNA processing. Bar plots represent enrichment significance expressed as −log_10_ (adjusted p-value).

Functional enrichment analysis was performed on 596 significantly dysregulated transcripts (FC ≥ 2.0, p < 0.05) identified in TQ-treated cells. Overrepresentation analysis using g:Profiler revealed significant enrichment of Gene Ontology Biological Process categories primarily related to cell cycle regulation, mitotic progression, chromosome organization, and ribosome biogenesis (adjusted p < 0.05) (Figure 3A).

Reactome pathway analysis further supported these findings, identifying significant enrichment of pathways associated with mitotic prometaphase, RHO GTPase signaling, mRNA splicing, and RNA metabolism (adjusted p < 0.05) (Figure 3B).

Collectively, these results indicate that TQ reshapes signaling networks governing proliferation, cytoskeletal organization, and cellular adaptation.

### 3.3 5-Fluorouracil

**FIGURE 4:**
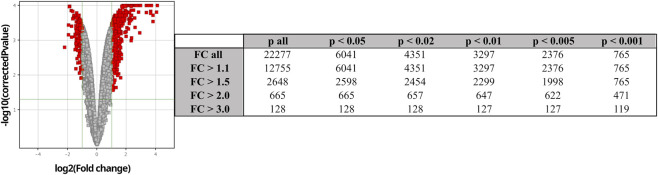
Volcano plot of differential gene expression in RKO colorectal cancer cells following 5-fluorouracil treatment compared with control cells. Differentially expressed transcripts are highlighted in red. The accompanying table summarizes the number of transcripts meeting defined fold change (FC) and statistical significance thresholds.

Transcriptomic profiling revealed marked changes in transcript expression following 5-fluorouracil treatment compared with untreated controls. A total of 665 transcripts were significantly differentially expressed at a fold change threshold of FC ≥ 2.0 (p < 0.05), including 128 transcripts showing pronounced regulation exceeding FC ≥ 3.0 (Figure 4).

As observed for thymoquinone, the number of differentially expressed transcripts decreased with increasing fold change thresholds. However, 5-FU treatment produced a larger subset of strongly dysregulated transcripts, consistent with a robust cytotoxic and stress-associated transcriptional response.

**FIGURE 5:**
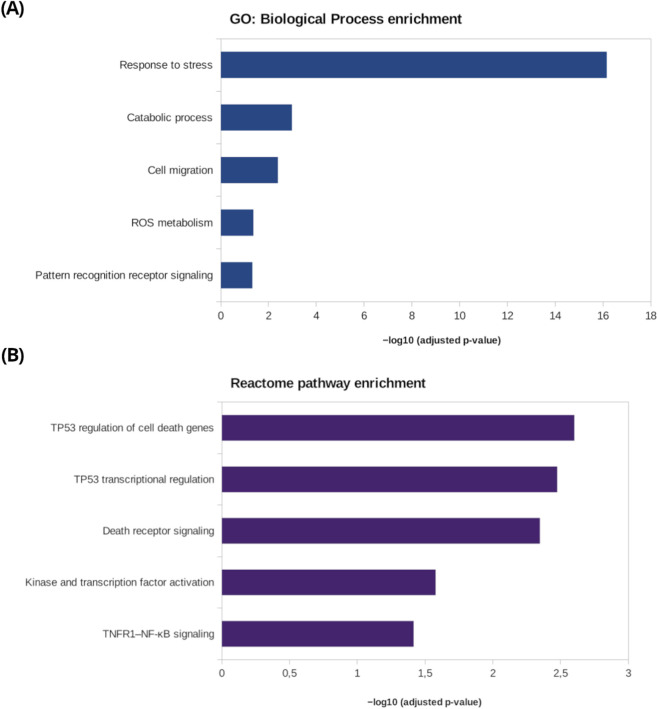
Functional enrichment analysis of transcripts upregulated in RKO colorectal cancer cells following 5-fluorouracil treatment. **(A)** Significantly enriched Gene Ontology (GO) Biological Process categories. **(B)** Significantly enriched Reactome pathways, highlighting stress response, TP53 signaling, and death receptor–related pathways. Bar plots represent enrichment significance expressed as −log_10_ (adjusted p-value).

Upregulated transcripts in 5-FU-treated RKO cells were predominantly enriched in biological processes related to cellular stress response, reactive oxygen species metabolism, and catabolic processes (Figure 5A). Pathway analysis further revealed significant overrepresentation of TP53-regulated transcriptional programs, death receptor signaling, and TNFR1/NF-κB signaling pathways (Figure 5B).

Collectively, these findings indicate that 5-FU induces a robust stress-associated transcriptional program linked to apoptotic and adaptive signaling responses.

**FIGURE 6:**
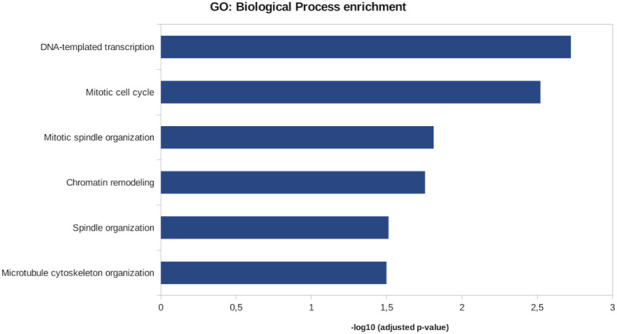
Functional enrichment analysis of transcripts downregulated in RKO colorectal cancer cells following 5-fluorouracil treatment. Significantly enriched Gene Ontology (GO) Biological Process categories are shown, highlighting pathways related to mitotic progression, spindle organization, chromatin remodeling, and transcriptional regulation. Bar plots represent enrichment significance expressed as −log_10_ (adjusted p-value).

Functional enrichment analysis of transcripts downregulated following 5-fluorouracil treatment revealed significant overrepresentation of biological processes related to mitotic cell cycle progression, cell division, spindle organization, chromatin organization, and DNA-templated transcription (adjusted p < 0.05) (Figure 6). These findings indicate suppression of proliferative and transcriptional programs in response to 5-FU exposure.

Reactome pathway analysis additionally identified enrichment of pathways associated with EIF2AK1-mediated stress signaling and formation of senescence-associated heterochromatin foci, supporting induction of integrated stress responses, senescence-associated remodeling, and adaptive chromatin reprogramming (not shown).

### 3.4 Combined treatment

Global transcriptomic profiling of RKO cells exposed to combined thymoquinone and 5-fluorouracil treatment revealed substantial alterations in transcript expression. Of the 22,277 analyzed transcripts, 3,763 showed statistically significant differential expression (p < 0.05).

**FIGURE 7:**
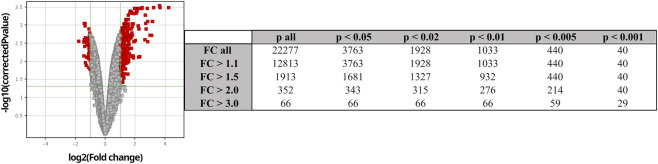
Volcano plot of differential gene expression in RKO colorectal cancer cells following combined thymoquinone and 5-fluorouracil treatment compared with control cells. Differentially expressed transcripts are highlighted in red. The accompanying table summarizes the number of transcripts meeting defined fold change (FC) and statistical significance thresholds.

Application of increasingly stringent fold change thresholds progressively reduced the number of regulated transcripts (Figure 7). At FC ≥ 2.0, 343 transcripts remained significantly differentially expressed, while 66 transcripts exceeded FC ≥ 3.0, including 59 with p < 0.005 and 29 with p < 0.001.

Compared with single-agent treatments, the combination induced a more selective subset of strongly regulated genes, suggesting coordinated rewiring of shared signaling pathways rather than generalized transcriptional amplification.

**FIGURE 8:**
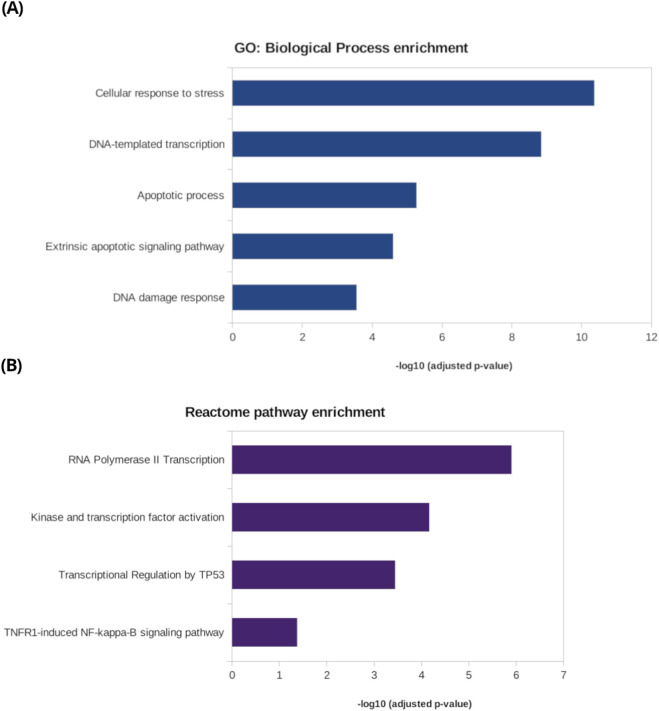
Functional enrichment analysis of transcripts upregulated in RKO colorectal cancer cells following combined thymoquinone and 5-fluorouracil treatment. **(A)** Significantly enriched Gene Ontology Biological Process categories. **(B)** Significantly enriched Reactome pathways, highlighting stress response, apoptotic signaling, TP53 regulation, and TNFR1/NF-κB–related pathways. Bar plots represent enrichment significance expressed as −log_10_ (adjusted p-value).

Functional enrichment analysis of transcripts upregulated following combined TQ and 5-FU treatment revealed significant overrepresentation of biological processes related to regulation of apoptosis, extrinsic apoptotic signaling, DNA damage response, and cellular stress response (adjusted p < 0.05). Additional enrichment of terms associated with regulation of gene expression and RNA polymerase II–mediated transcription suggests coordinated activation of transcriptional regulatory programs (Figure 8A).

Reactome pathway analysis further identified significant enrichment of TP53-mediated transcriptional regulation and TNFR1-induced NF-κB signaling pathways, supporting activation of interconnected stress-, survival-, and apoptosis-related signaling cascades in response to combination treatment (Figure 8B). In contrast, downregulated transcripts did not show significant enrichment of specific GO Biological Process or Reactome categories. These findings suggest that combined treatment promotes a coordinated cell fate response rather than simple additive cytotoxicity.

### 3.5 Shared transcriptional response to TQ, 5-FU, and combination treatment

To identify genes commonly regulated across all treatment conditions, overlap analysis was performed using significantly differentially expressed transcripts (FC ≥ 2.0, p < 0.05).

**FIGURE 9:**
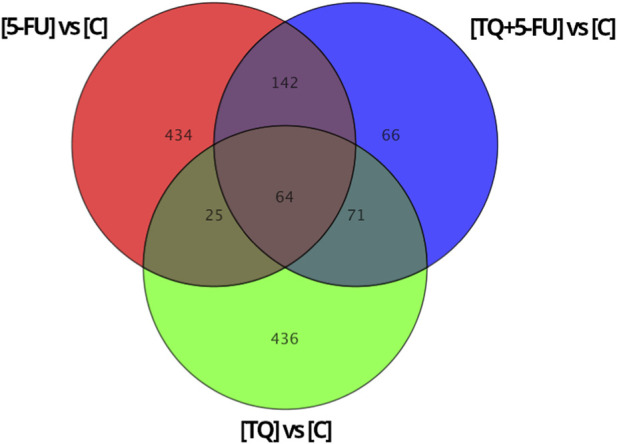
Venn diagram showing the overlap of significantly differentially expressed transcripts (FC ≥ 2.0, p < 0.05) in RKO colorectal cancer cells following thymoquinone [TQ], 5-fluorouracil [5-FU], and combined treatment [TQ+5-FU], each compared with untreated control cells [C]. A total of 64 transcripts were commonly regulated across all treatment conditions and used to define the core treatment-responsive gene signature.

Venn diagram analysis revealed 64 transcripts shared among thymoquinone, 5-fluorouracil, and combination treatment groups (Figure 9). The complete list of shared transcripts is presented in Supplementary Table S2.

When multiple probe sets corresponded to the same gene symbol, redundant entries were consolidated by retaining the probe set with the highest absolute fold change value. Following this filtering step, 54 unique genes were retained for subsequent functional and network analyses, representing a core transcriptional signature consistently modulated across all treatment conditions.

This shared gene set suggests convergence of distinct treatments on common pathways governing cell fate decisions.

The shared gene set included functionally relevant clusters associated with apoptosis/cell fate (*FAS, BIRC2, BIRC3*), stress response (*DNAJB4, HSPA4L, FOS*), and signaling or transcriptional regulation (*FZD6, MYBL1, EIF5*), supporting convergence on common adaptive and cell death–related pathways.

Functional enrichment analysis of the 54 shared genes revealed significant overrepresentation of pathways associated with necroptosis and TNF-mediated signaling (Table 3). KEGG analysis further identified enrichment of the TNF signaling pathway, apoptosis, and Hippo signaling, whereas Reactome highlighted TNFR1-induced pro-apoptotic signaling and kinase-dependent transcriptional activation. Collectively, these findings indicate convergence of all treatment conditions on TNF-centered cell death signaling and stress-responsive transcriptional programs.

**TABLE 3:** Functional enrichment analysis of the shared gene signature converging across thymoquinone (TQ), 5-fluorouracil (5-FU), and combined treatment conditions. Representative genes indicate selected genes contributing to enrichment of each pathway term.

Term	Adjusted p-value	Representative genes
GO: Biological process		
Necroptotic process	9.13 × 10^−5^	*BIRC2,BIRC3,FAS,CYLD*
Ripoptosome assembly	4.70 × 10^−3^	*BIRC2,CYLD*
KEGG pathways		
TNF signaling pathway	9.13 × 10^−5^	*BIRC2,MAP3K8,FOS,BIRC3,FAS,CYLD*
Apoptosis	4.37 × 10^−3^	*BIRC2,FOS,BIRC3,FAS,EIF2AK3*
Hippo signaling pathway	8.93 × 10^−3^	*ID2,BIRC2,FZD6,STK3,BIRC3*
Reactome		
Nuclear events (kinase and transcription factor activation)	1.61 × 10^−2^	*ID2,EGR2,FOS,ARC*
TNFR1-induced proapoptotic signaling	2.67 × 10^-^	*BIRC2,BIRC3,CYLD*

To assess transcriptomic interactions between thymoquinone and 5-fluorouracil, interaction terms were estimated using a deviation-from-additivity model based on log_2_ fold change values. The analysis revealed predominantly negative interaction values across the shared gene set, indicating that the combined treatment generally did not exceed the expected additive response derived from individual drug responses.

However, although the combined treatment generally did not exceed the expected additive transcriptional response, a subset of genes displayed higher logFC values in the combination group than in at least one single-agent treatment, and in several cases the combined treatment produced the strongest overall transcriptional response.

These findings suggest selective enhancement of treatment-responsive genes within a sub-additive global framework, indicating that combined treatment primarily reconfigures transcriptional response networks rather than uniformly amplifying transcript abundance in a synergistic manner.

### 3.6 Interaction network analysis and clinical relevance of selected genes

**FIGURE 10:**
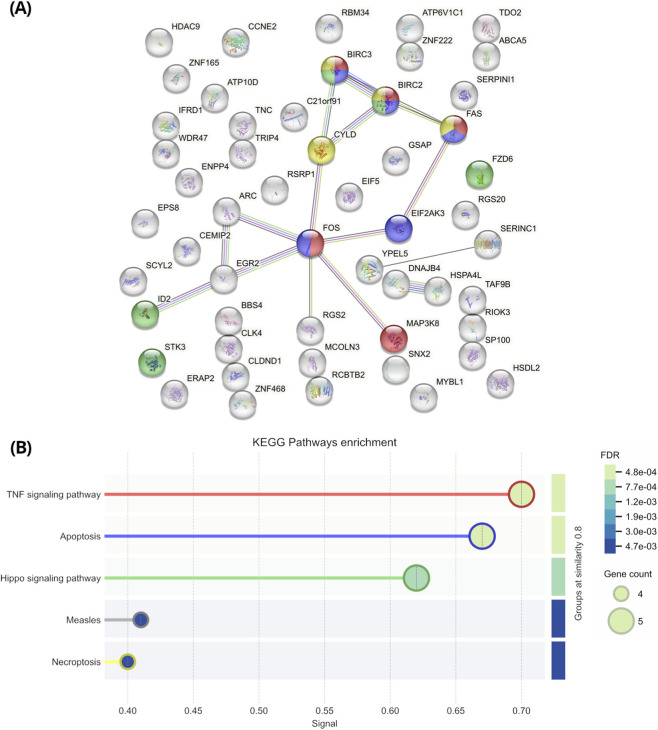
Interaction network and pathway enrichment analysis of the shared gene signature in RKO colorectal cancer cells following thymoquinone, 5-fluorouracil, and combined treatment. **(A)** Protein–protein interaction network highlighting key hub genes, including BIRC2, BIRC3, FAS, CYLD, and FOS. **(B)** KEGG pathway enrichment analysis demonstrating significant clustering within TNF signaling, apoptosis, necroptosis, and Hippo pathways.

The constructed protein–protein interaction network comprised 53 nodes and 16 edges, exceeding the expected number of interactions for a random gene set of comparable size (expected edges = 10; PPI enrichment p < 0.05). These findings indicate that the shared genes form a biologically interconnected functional module rather than a random collection of transcripts (Figure 10A).

Central nodes within the network included *BIRC2, BIRC3, FAS, CYLD*, and *FOS,* representing key regulators of TNF-mediated apoptotic and necroptotic signaling. KEGG pathway mapping further demonstrated clustering of proteins involved in TNF signaling, apoptosis, necroptosis, and Hippo pathways, supporting the functional coherence of the identified transcriptional signature (Figure 10B).

Overall, the observed network connectivity indicates coordinated regulation of cell death–associated signaling pathways across treatment conditions. These central nodes define a TNF-centered regulatory axis governing the balance between cell survival and programmed cell death.

Based on network topology and pathway associations, *BIRC2, BIRC3, FAS, CYLD, EIF2AK3, ID2, MAP3K8, FOS, STK3*, and *FZD6* were selected for subsequent validation and clinical correlation analyses.

**FIGURE 11:**
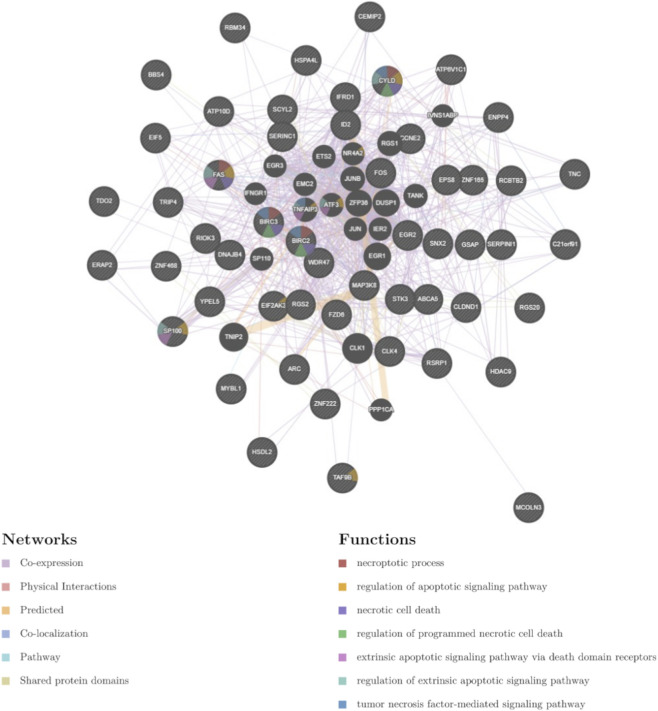
Gene interaction network of the shared gene signature generated using GeneMANIA. Nodes represent genes, and edges indicate functional associations based on co-expression, physical interactions, predicted interactions, co-localization, pathway co-membership, and shared protein domains. Key genes, including *BIRC2, BIRC3, FAS, CYLD*, and *EIF2AK3*, form a central cluster associated with TNF signaling, apoptosis, and necroptosis. Colored segments within nodes denote predominant functional annotations related to cell death–associated processes, highlighting a coordinated, TNF-centered regulatory network underlying treatment-induced cell fate responses.

To further investigate functional relationships among the shared genes, network analysis was performed using GeneMANIA. The resulting network demonstrated extensive functional connectivity, supported predominantly by co-expression, pathway co-membership, and shared protein domain evidence (Figure 11).

A prominent functional cluster was observed around key regulators of cell death–related signaling, including *BIRC2, BIRC3, CYLD, FAS*, and *EIF2AK3.* Several of these genes were associated with multiple enriched pathways, including TNF signaling, apoptosis, and necroptosis, indicating coordinated regulation of cell death–related processes. These findings further support the biological coherence of the identified transcriptional signature and reinforce the selection of central network components for subsequent analyses.

Collectively, these results support the presence of a coordinated, TNF-centered regulatory network governing treatment-induced cell fate decisions.

UALCAN analysis was performed to assess the clinical relevance of selected candidate genes in colorectal cancer tissues using TCGA datasets. Genes were selected from the microarray dataset based on significant differential expression (FC ≥ 2.0) and their involvement in key pathways related to programmed cell death and TNF-mediated signaling, as identified in functional enrichment analyses. Several of these genes were also included in subsequent RT-qPCR validation due to their potential regulatory roles in apoptosis, necroptosis, and inflammatory signaling.

**FIGURE 12:**
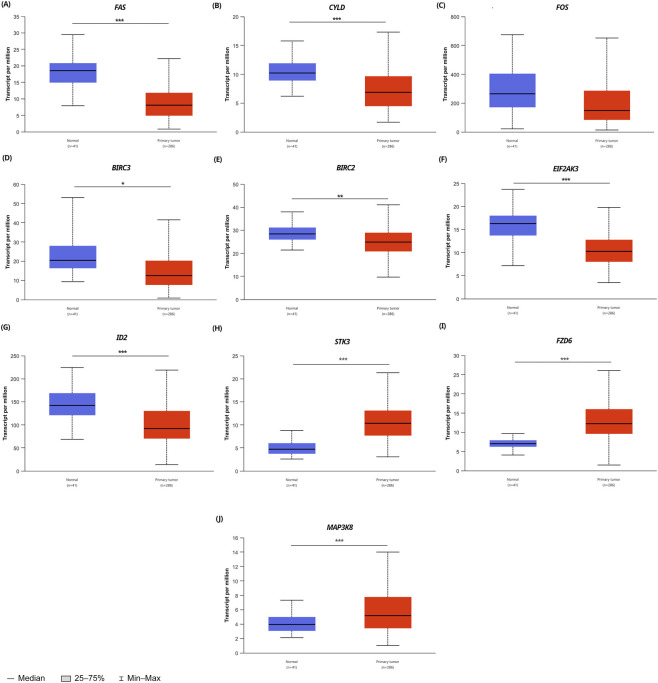
Expression levels of selected cell death- and stress-related genes in colorectal cancer tissues compared with normal colon samples based on UALCAN/TCGA analysis. Panels show: **(A)** *FAS*, **(B)** *CYLD*, **(C)** *FOS*, **(D)** *BIRC3*, **(E)** *BIRC2*, **(F)** *EIF2AK3*, **(G)** *ID2*, **(H)** *STK3*, **(I)** *FZD6*, and **(J)** *MAP3K8*. Data are presented as transcripts per million. Box plots represent gene expression levels in normal and tumor tissues. Genes involved in cell death and TNF-related signaling (*FAS, CYLD, EIF2AK3, ID2, BIRC2, BIRC3*) are predominantly downregulated in tumor samples, whereas STK3, FZD6, and MAP3K8 show increased expression. Statistical significance is indicated as follows: *p < 0.05, **p < 0.01, ***p < 0.001. These patterns indicate suppression of cell death–related pathways and concurrent activation of pro-survival signaling in colorectal cancer tissues.

The analysis revealed that key regulators of cell death pathways, including *FAS, CYLD, EIF2AK3*, *ID2, BIRC2*, and *BIRC3,* were significantly downregulated in colorectal adenocarcinoma tissues compared with normal colon samples. In contrast, *STK3, FZD6*, and *MAP3K8* were upregulated in tumor tissues, while no significant differences in *FOS* expression were observed between tumor and normal samples (Figure 12).

Notably, treatment of colorectal cancer cells with thymoquinone, 5-fluorouracil, or their combination resulted in increased expression of all selected genes compared with untreated controls. These findings suggest that the applied treatments may partially restore the activity of cell death–and TNF-related signaling pathways that are suppressed during colorectal cancer progression. These patterns indicate suppression of cell death–related pathways and concurrent activation of pro-survival signaling in colorectal cancer tissues.

### 3.7 RT-qPCR validation of selected genes

**FIGURE 13:**
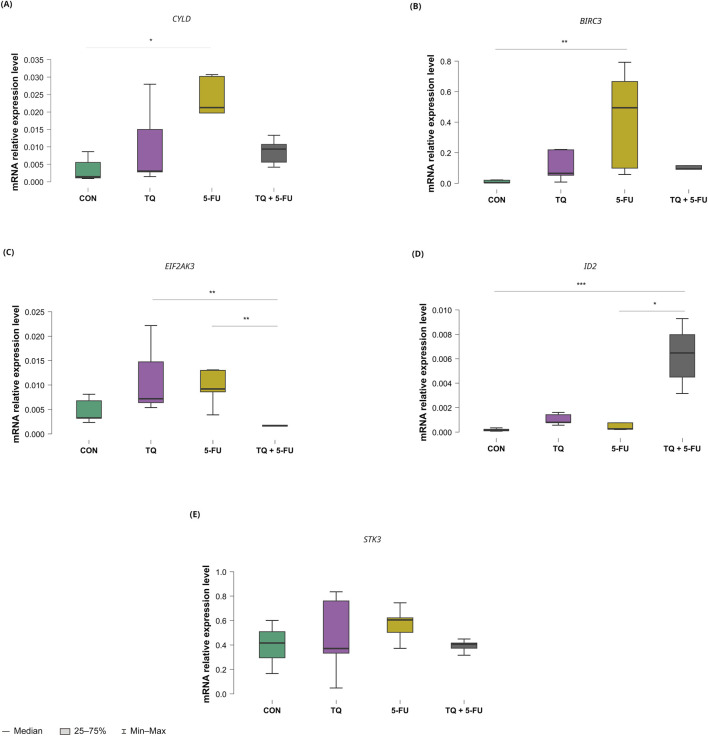
RT-qPCR validation of selected genes in RKO colorectal cancer cells following treatment with thymoquinone (TQ), 5-fluorouracil (5-FU), or their combination (TQ+5-FU). Relative mRNA expression levels of *CYLD* **(A)**, *BIRC3* **(B)** *, EIF2AK3* **(C)** *, ID2* **(D)**, and *STK3* **(E)** are shown. Data are presented as median with interquartile range (IQR). Statistical significance was determined using the Kruskal–Wallis test followed by *post hoc* pairwise comparisons (*p < 0.05; **p < 0.01; ***p < 0.001).

Based on the microarray analysis, *CYLD, BIRC3, EIF2AK3, ID2*, and *STK3* were selected for RT-qPCR validation due to their significant differential expression (FC ≥ 2.0) and involvement in key signaling pathways associated with programmed cell death and TNF-mediated signaling (Figure 13). These genes were considered representative components of the identified transcriptional signature, encompassing regulators of apoptosis, stress response, and signaling pathways implicated in cell fate determination. Due to the exploratory transcriptomic nature of the study, RT-qPCR validation was performed for a representative subset of genes rather than all differentially expressed candidates identified in the microarray and network analyses.

*CYLD* expression was significantly increased in RKO cells treated with 5-FU compared with untreated controls (p < 0.05), consistent with the microarray data (FC = 4.91). Similarly, *BIRC3* expression was significantly upregulated following 5-FU treatment (p < 0.01), confirming the transcriptomic findings. No statistically significant differences were observed for these genes under other treatment conditions despite fold changes exceeding 2.0 in the microarray analysis.

*EIF2AK3* expression did not differ significantly from control levels in any treatment group, despite increased expression detected in the microarray dataset. However, expression in the combination group was significantly reduced compared with both monotherapies (p < 0.01).

*ID2* expression was significantly increased exclusively in the combination treatment group (p < 0.001), in agreement with the microarray results. In contrast, the elevated expression observed in TQ and 5-FU monotherapies at the microarray level was not confirmed by RT-qPCR.

No statistically significant differences in *STK3* expression were detected between groups, likely reflecting the relatively modest fold changes observed in the microarray analysis.

Overall, RT-qPCR validation confirmed key microarray findings while also revealing gene- and treatment-specific differences in expression patterns. Although the general direction of expression changes was consistent for several analyzed genes, discrepancies in expression magnitude and statistical significance were observed for selected targets. Such differences are relatively common in transcriptomic studies and may result from methodological differences between microarray hybridization and PCR-based transcript quantification, including differences in sensitivity, dynamic range, probe specificity, normalization procedures, amplification efficiency, and transcript abundance. In addition, biological variability and relatively modest fold changes observed for selected genes may further contribute to differences between both analytical platforms.

### 3.8 Western blot analysis of Fas expression

**FIGURE 14:**
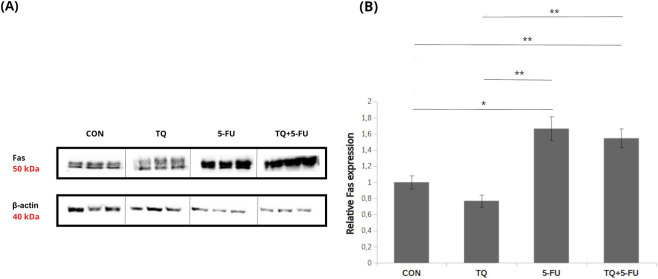
Western blot analysis of Fas protein expression in RKO colorectal cancer cells following treatment with thymoquinone (TQ), 5-fluorouracil (5-FU), or their combination (TQ+5-FU). **(A)** Representative immunoblots of Fas and β-actin. **(B)** Densitometric quantification of Fas expression normalized to β-actin. Images were cropped for clarity; non-adjacent lanes are indicated by vertical lines. Data are presented as mean ± SD from three independent experiments (n = 3). Statistical significance was determined using one-way ANOVA followed by Tukey’s *post hoc* test (*p < 0.05; **p < 0.01).

Western blot analysis revealed differential regulation of Fas protein expression across treatment conditions (Figure 14). Full-length, uncropped Western blot images are shown in Supplementary Figure S1. Densitometric quantification normalized to β-actin demonstrated a significant overall effect of treatment on Fas protein expression (one-way ANOVA, F (3,8) = 20.11, p = 0.00044), which was primarily associated with increased Fas levels in the 5-FU and TQ+5-FU treatment groups.

Treatment with 5-FU significantly increased Fas expression (1.67-fold vs. control), as did the combined TQ+5-FU treatment (1.54-fold vs. control). In contrast, TQ alone did not significantly alter Fas protein levels (0.77-fold vs. control).

Post hoc Tukey’s test confirmed significant differences between control and 5-FU-treated cells, as well as between control and the combination group. Moreover, both 5-FU and TQ+5-FU treatments differed significantly from TQ alone, whereas no significant difference was observed between 5-FU and TQ+5-FU groups.

These results indicate that 5-FU is the primary driver of Fas protein upregulation, whereas TQ alone does not independently induce Fas expression at the protein level. However, TQ also does not attenuate the effect of 5-FU in the combination setting. Importantly, the potential modulatory effect of TQ on treatment response is unlikely to depend exclusively on direct regulation of FAS expression, but rather on coordinated reprogramming of multiple interconnected stress- and cell death–related signaling pathways identified in the transcriptomic analyses.

**FIGURE 15:**
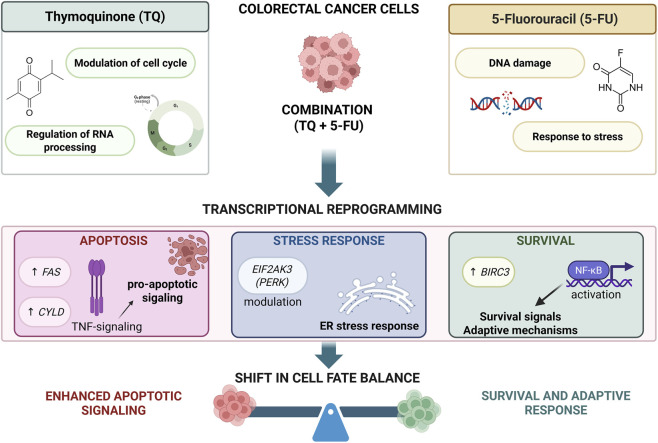
Network-level modulation of 5-fluorouracil response by thymoquinone in colorectal cancer cells. Schematic representation of the transcriptional reprogramming induced by thymoquinone (TQ), 5-fluorouracil (5-FU), and their combination in colorectal cancer cells. TQ primarily modulates cell cycle progression and RNA processing, whereas 5-FU induces DNA damage and activates cellular stress responses. Combined treatment results in coordinated transcriptional reprogramming, affecting key signaling pathways involved in apoptosis (↑*FAS*, ↑*CYLD*), stress response (*EIF2AK3/PERK*), and survival mechanisms *(↑BIRC3*, NF-κB activation). These interactions reflect network-level modulation rather than classical transcriptional synergy. The integration of these pathways leads to a shift in the balance between pro-apoptotic signaling and adaptive survival responses, highlighting the role of TQ as a modulator of chemotherapy response. Created in BioRender. Kurowska et al. (2026) https://BioRender.com/n2pwa3d Created with BioRender.com.

To summarize the observed transcriptional changes and their functional implications, a schematic model of the proposed mechanism underlying the interaction between thymoquinone and 5-fluorouracil is presented (Figure 15). This model highlights network-level reprogramming of cell death pathways rather than classical transcriptional synergy.

## 4 Discussion

Systemic treatment of colorectal cancer remains largely dependent on fluoropyrimidine-based chemotherapy, particularly 5-fluorouracil which constitutes the backbone of widely used regimens such as FOLFOX and FOLFIRI (Morris et al., 2023). Despite decades of clinical use and well-established efficacy, these agents act through highly cytotoxic and largely non-selective mechanisms, leading to substantial damage to normal tissues. Consequently, fluoropyrimidine-based therapies are frequently associated with adverse effects that compromise patients’ quality of life and may ultimately limit therapeutic efficacy (Soveri et al., 2014).

In parallel, increasing attention has been directed toward bioactive compounds derived from natural products, many of which are being actively investigated for their anticancer potential. Among these, thymoquinone, the principal bioactive constituent of *N. sativa*, has attracted considerable interest due to its pleiotropic biological activities (Kurowska et al., 2023). Numerous studies have demonstrated that TQ exerts antiproliferative and pro-apoptotic effects across multiple cancer models, including colorectal cancer, while exhibiting relatively low toxicity toward normal cells (Al-Damook et al., 2025).

These properties suggest that TQ may function not only as a cytotoxic agent but also as a modulator of signaling pathways relevant to cancer cell survival and stress response. However, despite accumulating evidence supporting its anticancer activity, relatively few studies have investigated the combined effects of TQ and 5-FU in colorectal cancer, and the system-level molecular mechanisms underlying such interactions remain insufficiently characterized.

In this context, the present study aimed to characterize the global transcriptional response to TQ, 5-FU, and their combination, with particular emphasis on network-level regulation of cell death pathways.

We investigated the transcriptional response of colorectal cancer cells to thymoquinone, 5-fluorouracil, and their combination using a microarray-based approach, complemented by *in silico* analyses and experimental validation at both the mRNA and protein levels. Our results revealed extensive modulation of gene expression across all treatment conditions, with a subset of genes consistently regulated, suggesting convergence on shared signaling pathways.

Thymoquinone treatment was primarily associated with transcriptional changes related to cell cycle regulation and mitotic progression. Enrichment of pathways linked to chromosome organization, mitotic division, and RNA processing suggests that TQ induces a coordinated reprogramming of cellular processes governing proliferation and biosynthetic activity. Rather than directly activating classical cell death pathways, these changes likely reflect disruption of mitotic homeostasis and induction of a stress-adaptive state.

From a mechanistic perspective, such transcriptional reprogramming may reflect modulation of apoptosis and stress-related signaling pathways involved in regulation of colorectal cancer cell fate. These findings support the notion that TQ acts primarily as a modulator of cellular signaling networks rather than exerting direct cytotoxic effects.

In contrast to thymoquinone, the transcriptional profile induced by 5-fluorouracil reflects a robust activation of cellular stress responses associated with oxidative damage and catabolic processes. The enrichment of pathways related to reactive oxygen species metabolism and cellular stress is consistent with the well-established mechanism of 5-FU, which involves disruption of nucleotide metabolism and induction of DNA damage.

Pathway analysis further highlighted activation of TP53-dependent transcriptional programs, death receptor signaling, and TNFR1–NF-κB pathways, all of which play central roles in stress-mediated cell fate regulation. Concurrently, genes downregulated following 5-FU exposure were predominantly associated with mitotic progression, including *PLK1* (FC = **−**2.27)*, CDC20* (FC = **−**2.09)*, MYC* (FC = **−**2.14), and *CCND1* (FC = **−**2.65), as well as chromatin organization related genes such as *ATRX* (FC = **−**2.05)*, SMARCA4* (FC = **−**2.06), and *HMGA2* (FC = **−**2.43). In addition, altered expression of transcriptional regulators including *ATF4* (FC = **−**2.21)*, FOSL1* (FC = **−**2.16)*, E2F5* (FC = **−**2.10), and *ZEB1* (FC = **−**2.57) indicated suppression of proliferative activity and global biosynthetic programs.

Additional enrichment of pathways related to EIF2AK1-mediated stress signaling and the formation of senescence-associated heterochromatin foci suggests engagement of translational control mechanisms and potential induction of a senescence-like state.

Collectively, these findings indicate that 5-FU induces a coordinated stress response characterized by activation of cell death–related signaling pathways alongside suppression of proliferation, consistent with its cytotoxic mode of action.

Notably, combined treatment with thymoquinone and 5-fluorouracil revealed a transcriptional pattern consistent with coordinated activation of stress- and cell death–related signaling pathways. In contrast to individual treatments, the combined exposure preferentially engaged biological processes associated with apoptosis, extrinsic apoptotic signaling, DNA damage response, and broader cellular stress programs.

Enrichment of transcription-related processes, including RNA polymerase II–mediated gene expression, further suggests that the combined treatment induces coordinated transcriptional reprogramming rather than isolated pathway activation. Consistently, pathway analysis highlighted the involvement of TP53-dependent networks and TNFR1–NF-κB signaling, both of which are central regulators of stress-mediated cell fate decisions.

These findings indicate that simultaneous exposure to TQ and 5-FU reinforces convergent signaling cascades governing apoptosis and stress adaptation. Importantly, this effect appears to arise from integration of overlapping pathways rather than classical synergistic amplification.

From a biological perspective, such coordinated activation of multiple cell death–related pathways is particularly relevant in colorectal cancer, where resistance to apoptosis represents a major barrier to effective therapy. Increasing evidence suggests that simultaneous engagement of distinct regulated cell death mechanisms may represent an effective strategy to overcome adaptive resistance and enhance anti-tumor responses. Supporting this concept, Yang et al. (2026) demonstrated that glutathione depletion induced concurrent activation of ferroptosis and cuproptosis in hepatocellular carcinoma, highlighting the therapeutic relevance of coordinated activation of multiple cell death programs across different tumor contexts. In this context, TQ may enhance the anticancer activity of 5-FU by modulating the transcriptional landscape and increasing susceptibility to stress-induced cell death. Collectively, these findings support the concept that TQ functions as a network-level modulator of chemotherapy response, promoting transcriptional reprogramming of signaling pathways that regulate the balance between cell survival and cell death.

Importantly, interaction analysis of the 54 genes commonly modulated across all treatment groups did not reveal widespread transcriptional responses exceeding the additive expectation as interaction terms were predominantly negative. Accordingly, negative interaction values should not be interpreted as pharmacological antagonism or suppression of 5-FU activity. Instead, they may reflect overlapping signaling pathways, compensatory regulatory mechanisms, or network-level transcriptional reprogramming in response to combined treatment. Consequently, reduced expression of selected genes in the TQ+5-FU group relative to 5-FU monotherapy likely reflects redistribution and reorganization of treatment-induced signaling networks, resulting in coordinated modulation of stress-, apoptosis-, and TNF-related pathways. However, the absence of transcriptional responses exceeding the additive expectation does not imply the absence of biologically meaningful interaction. For a subset of shared genes, the combined treatment produced higher expression changes than at least one of the individual treatments, indicating selective enhancement of transcriptional responses. This framework therefore enables quantitative assessment of transcriptional interactions at the systems level while acknowledging that transcriptional deviations from additivity do not necessarily correspond to pharmacological synergy or antagonism.

Moreover, the combination of TQ and 5-FU generated a distinct transcriptional profile within the shared gene set, suggesting convergence on overlapping signaling pathways accompanied by network-level reprogramming of stress and cell death responses. In this context, the observed transcriptional rewiring likely reflects a coordinated cellular adaptation to combined treatment, whereby TQ modulates the molecular response to 5-FU rather than uniformly amplifying gene expression. This supports the concept that TQ acts as a context-dependent regulator of chemotherapy response, reshaping signaling networks that determine cell fate.

To further investigate functional relationships among genes commonly modulated across all treatment conditions, network-based *in silico* analyses were performed focusing on the subset of 54 shared genes. Protein–protein interaction analysis using STRING and gene interaction network reconstruction with GeneMANIA revealed a highly interconnected network enriched in regulators of apoptosis, stress signaling, and transcriptional control. Within this network, several genes emerged as key regulatory nodes, including *FAS, CYLD, FOS, BIRC2, BIRC3, STK3, FZD6, MAP3K8, ID2,* and *EIF2AK3*. Their prominent positioning within the interaction network, together with consistent transcriptional modulation, suggests an important role in treatment-induced cellular responses.

Notably, many of these genes are functionally linked to TNF-mediated signaling and cell death pathways, suggesting the presence of a coordinated regulatory axis integrating apoptotic, necroptotic, and stress-response mechanisms. These candidates were therefore prioritized for further analysis based on their network connectivity and biological relevance within the identified transcriptional landscape.

Among these targets, FAS was selected for protein-level validation by Western blot analysis because it represented a key component of the identified TNF-related apoptotic signaling network and demonstrated consistent regulation across transcriptomic, network, and clinical expression analyses.

*FAS* expression is significantly downregulated in colorectal cancer compared with normal colonic mucosa, as confirmed by UALCAN analysis, consistent with its established role in tumor progression and immune evasion. Normal colonic epithelial cells constitutively express the Fas receptor and remain sensitive to FasL-induced apoptosis (O’Connell et al., 2000), whereas loss of Fas observed in a substantial proportion of colorectal tumors represents an important mechanism of apoptotic resistance (Ogawa et al., 2004).

In our previously published study (Kurowska et al., 2026), which focused on cytotoxicity and apoptosis-related responses in colorectal cancer cells, treatment with 5-fluorouracil alone or in combination with thymoquinone significantly increased *FAS* expression. In the present study, this effect was independently confirmed at both the transcriptomic and protein levels. These findings suggest that treatment-induced upregulation of *FAS* may partially restore apoptotic sensitivity in colorectal cancer cells, thereby enhancing responsiveness to cytotoxic therapy.

Although microarray analysis indicated a moderate increase in *FAS* transcript levels following TQ treatment, this change was not independently confirmed at the RT-qPCR or protein level. The discrepancy observed for TQ treatment may reflect differences in sensitivity between transcriptomic and targeted validation approaches, as well as post-transcriptional regulatory mechanisms affecting mRNA stability, translational efficiency, microRNA-mediated regulation, or protein turnover.

Therefore, the TQ-associated increase in *FAS* expression should be interpreted with caution and may represent a modest or transient transcriptional response that does not necessarily result in detectable protein accumulation under the experimental conditions used in this study.

*CYLD* expression is significantly reduced in colorectal cancer compared with normal colonic tissue, as confirmed by UALCAN analysis, consistent with its established role as a tumor suppressor regulating multiple signaling pathways (Hellerbrand et al., 2007).

In the present study, microarray analysis revealed increased *CYLD* expression across all treatment conditions (FC > 2), with the strongest effect observed following 5-fluorouracil exposure; these findings were further validated by RT-qPCR.

Functionally, *CYLD* acts as a negative regulator of NF-κB and Wnt/β-catenin signaling, and its restoration has been shown to suppress proliferation, invasion, and migration of colorectal cancer cells (Cheng et al., 2020; Yang et al., 2020). In this context, treatment-induced upregulation of *CYLD* may represent a key mechanism contributing to the observed antitumor effects, potentially restoring regulatory control over pro-survival signaling pathways and reinforcing apoptosis-related responses.

Based on TCGA data, *ID2* expression is reduced in colorectal cancer tissues compared with normal colonic mucosa. However, *ID2* exhibits a context-dependent role in colorectal cancer. In many experimental models, it functions as an oncogene promoting proliferation and survival, whereas in intestinal epithelial cells it may also exert tumor-suppressive effects (Gray et al., 2008; Russell et al., 2004).

Interestingly, protein-level analyses have reported elevated ID2 expression in colorectal tumors, suggesting a discrepancy between mRNA and protein levels that may be influenced by factors such as TP53 status, which differs between clinical samples and the RKO cell model (Wilson et al., 2001).

In our study, *ID2* expression was increased following treatment with 5-FU, TQ, and their combination, although this effect was only partially confirmed by RT-qPCR. Given its dual functional role, this upregulation likely reflects an adaptive cellular response to treatment-induced stress rather than a direct antitumor mechanism.

However, considering the reduced *ID2* mRNA levels observed in clinical datasets, treatment-induced upregulation may also indicate partial restoration of physiological regulatory functions in colorectal epithelial cells.

*BIRC3* also exhibits a context-dependent role in colorectal cancer. In our study, treatment with 5-FU led to a significant upregulation of *BIRC3* expression, which was confirmed by RT-qPCR. Although microarray data suggested increased *BIRC3* expression following TQ treatment and combination therapy, these effects were not consistently validated.

Analysis of TCGA datasets indicates that *BIRC3* expression is generally reduced in colorectal cancer tissues compared with normal controls; however, functional studies have reported elevated *BIRC3* levels in tumor cells, where it contributes to pro-survival signaling (Zhang et al., 2023).

Mechanistically, *BIRC3* is a well-established NF-κB target and inhibitor of apoptosis. Its induction by 5-FU may therefore represent a compensatory response to cytotoxic stress. Activation of NF-κB signaling can promote *BIRC3* expression, leading to suppression of caspase activity, attenuation of apoptosis, and enhanced chemoresistance (Frazzi, 2021). Recent evidence further highlights NF-κB signaling as a critical regulator of tumor cell survival and proliferation, suggesting that targeting NF-κB-related pathways may improve therapeutic efficacy and help overcome resistance mechanisms (Cheng et al., 2025). In this context, the observed upregulation of *BIRC3* may reflect a treatment-induced adaptive response that partially counteracts the pro-apoptotic effects of therapy. Such adaptive mechanisms are likely integrated within broader signaling networks rather than operating through isolated linear pathways. Consistent with this concept, previous studies have demonstrated that the PI3K/AKT/mTOR axis contributes to the regulation of colorectal cancer cell proliferation, survival, and apoptosis, underscoring the importance of interconnected pro-survival signaling pathways in shaping treatment responses (Tong et al., 2022).

Collectively, these observations identify *BIRC3* as a potential regulator of treatment response and a candidate contributor to chemoresistance in colorectal cancer.

*EIF2AK3 (PERK*) plays a context-dependent role in colorectal cancer, functioning as a key regulator of the unfolded protein response and cellular stress signaling. Activation of the *PERK*–ATF4 axis, for example, in response to 5-fluorouracil, has been associated with enhanced tumor cell survival and chemoresistance, whereas sustained or excessive activation may promote apoptosis (Nemati et al., 2025).

TCGA data indicate that *EIF2AK3* expression is reduced in colorectal cancer tissues compared with normal mucosa. In our study, combination treatment with TQ and 5-FU resulted in decreased *EIF2AK3* expression, as confirmed by RT-qPCR, partially diverging from the microarray data.

These findings highlight the complexity of PERK signaling in colorectal cancer, where both activation and inhibition of EIF2AK3 may exert context-dependent effects depending on the intensity and duration of cellular stress (Rozpędek et al., 2020; Shi et al., 2019). In this context, modulation of *EIF2AK3* by TQ may influence stress-response pathways, while its differential regulation under combination treatment may reflect a shift between adaptive and pro-apoptotic signaling states.

Taken together, *EIF2AK3* appears to function as a dynamic regulator of treatment response, underscoring the importance of balanced PERK signaling rather than its uniform activation or suppression.

*STK3* is a core component of the Hippo signaling pathway and is generally considered a tumor suppressor in colorectal cancer, with reduced expression associated with poor prognosis (Jung et al., 2023). However, emerging evidence suggests a more context-dependent role. For example*, STK3* has been identified as a potential transcriptional target of YAP1 and may participate in feedback regulation of oncogenic signaling pathways, including Wnt/β-catenin (Xie et al., 2025).

In our study, microarray analysis indicated a modest increase in *STK3* expression across all treatment conditions; however, these changes were not validated by RT-qPCR and were not statistically significant.

Taken together, while *STK3* may contribute to signaling crosstalk in colorectal cancer, our data do not support a major role for its modulation under the tested conditions, suggesting that its involvement in the observed treatment response is limited.

In summary, our study demonstrates that thymoquinone, 5-fluorouracil, and their combination induce distinct yet partially overlapping transcriptional programs in colorectal cancer cells, converging on pathways that regulate cellular stress, apoptosis, and cell fate determination. While TQ primarily modulates cell cycle–related and post-transcriptional processes, 5-FU elicits a robust stress response associated with DNA damage, TP53 activation, and suppression of proliferative programs.

Importantly, combined treatment promotes coordinated activation of multiple cell death–related pathways, reflecting network-level reprogramming rather than classical transcriptional synergy. Although no super-additive effects were observed, the combination generated a distinct transcriptional landscape, indicating functional interaction between both agents.

The identification of a shared gene signature and a TNF-centered regulatory network involving key genes such as *FAS, CYLD, BIRC3*, and *EIF2AK3* underscores the importance of integrated signaling pathways in shaping treatment response.

Collectively, these findings support the concept that TQ acts as a context-dependent modulator of chemotherapy response by reshaping signaling networks associated with cell death and stress adaptation rather than directly amplifying cytotoxic effects.

Importantly, the transcriptomic changes observed in the present study are consistent with our previously published findings obtained under comparable experimental conditions (Kurowska et al., 2026), in which TQ alone or in combination with 5-FU reduced RKO cell viability and induced apoptosis-related responses, including DNA fragmentation, caspase-3/7 activation, and increased expression of *FAS* and *BAX*.

Despite the comprehensive transcriptomic and network-based analyses presented in this study, several limitations should be acknowledged. First, the findings are based on a single colorectal cancer cell line (RKO), which may not fully capture tumor heterogeneity. Second, the lack of *in vivo* validation limits direct translation of these observations into a physiological or clinical context. An additional limitation of the present study is that the *in vitro* concentrations used may not directly reflect clinically achievable plasma exposure, particularly in the case of thymoquinone, for which pharmacokinetic and anticancer efficacy data in humans remain limited.

Future studies should therefore validate these findings in additional colorectal cancer models, including patient-derived systems, organoids, and *in vivo* models, to better reflect tumor heterogeneity and therapeutic response. Furthermore, given the identification of a TNF-centered regulatory network, targeted investigation of this signaling axis may provide deeper mechanistic insight and reveal novel opportunities for therapeutic intervention. Although transcriptomic and network analyses strongly implicated CYLD and NF-κB–related signaling in treatment response, the present study included protein-level validation only for FAS. Therefore, additional analyses investigating CYLD protein expression and NF-κB activation status are warranted to further define the mechanistic basis of the observed transcriptional reprogramming.

Such approaches may ultimately facilitate the development of more effective combination strategies targeting cell death–related pathways in colorectal cancer.

## 5 Conclusion

This study demonstrates that treatment with thymoquinone and 5-fluorouracil induces extensive transcriptional reprogramming of pathways regulating cell death, stress response, and proliferation in colorectal cancer cells. Rather than eliciting classical transcriptional synergy, the combined treatment reshapes cellular responses through coordinated modulation of overlapping signaling networks, particularly those involved in apoptosis and stress adaptation.

Key regulatory genes identified in this study, including *FAS* and *CYLD*, may contribute to enhanced pro-apoptotic signaling, whereas others, such as *BIRC3* and *EIF2AK3*, reflect adaptive or resistance-associated responses, highlighting the complexity of treatment-induced molecular dynamics.

Importantly, our findings indicate that TQ functions as a modulator of chemotherapy response rather than a direct cytotoxic agent, influencing the balance between survival and cell death pathways.

Collectively, these results support the potential of combining TQ with fluoropyrimidine-based chemotherapy as a strategy to improve therapeutic outcomes. However, further mechanistic studies and *in vivo* validation are required to confirm the clinical relevance of these findings (Lockhart et al., 1996; Nathanson et al., 2023).

## Data Availability

The microarray datasets are available in the GEO database under accession number GSE331234. Additional data supporting the findings of this study are included in the article and its Supplementary Material. Further information and materials are available from the corresponding author upon request.
