# Supplementary figures and images for: Network-level reprogramming of cell death pathways in colorectal cancer cells by combined thymoquinone and 5-fluorouracil treatment
Source: Front Mol Biosci. 2026 Jun 24;13:1864680. doi: 10.3389/fmolb.2026.1864680 (PMC13341502; doi:10.3389/fmolb.2026.1864680)

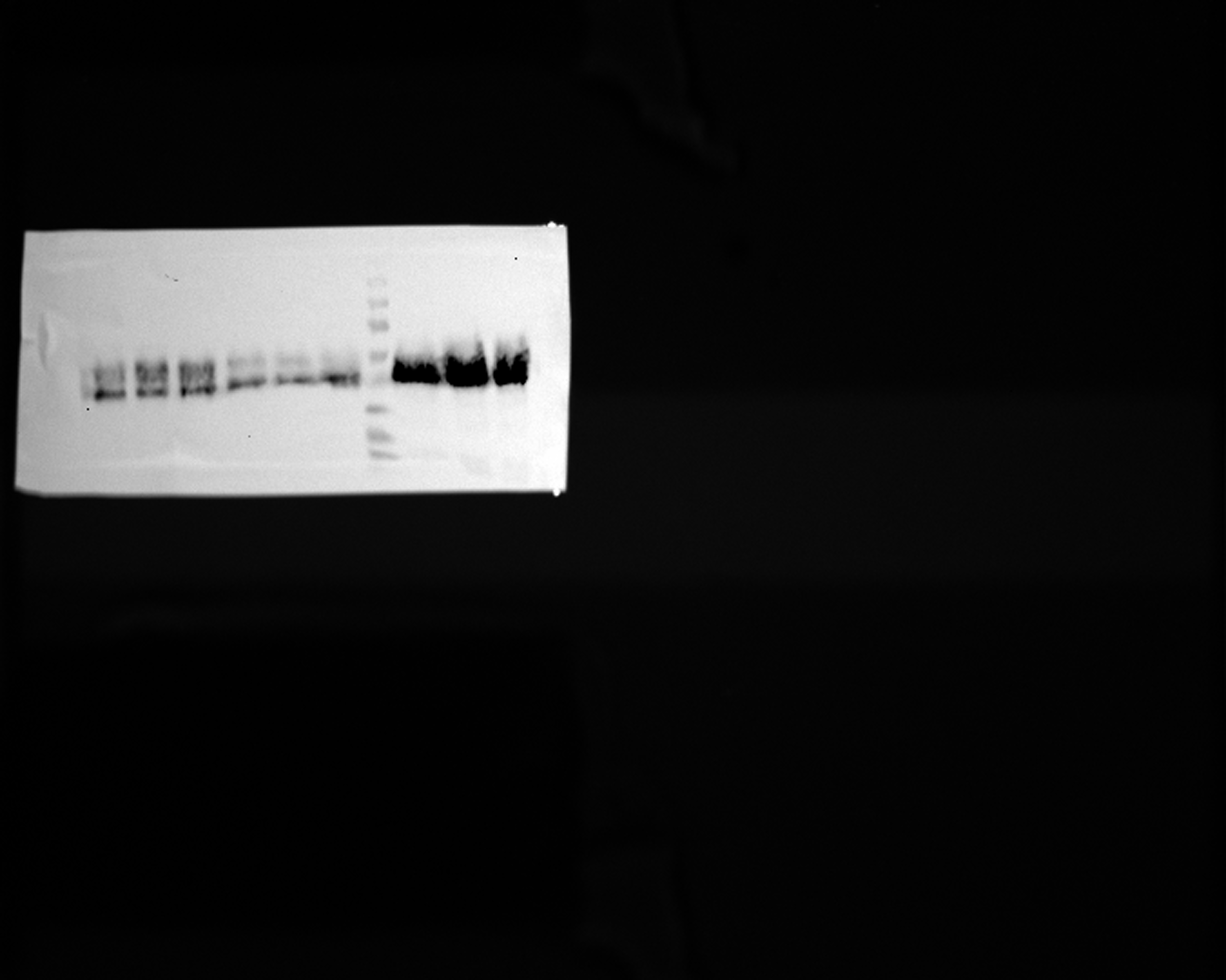

Supplement: Supplementary file 1 [file Image6.tif]

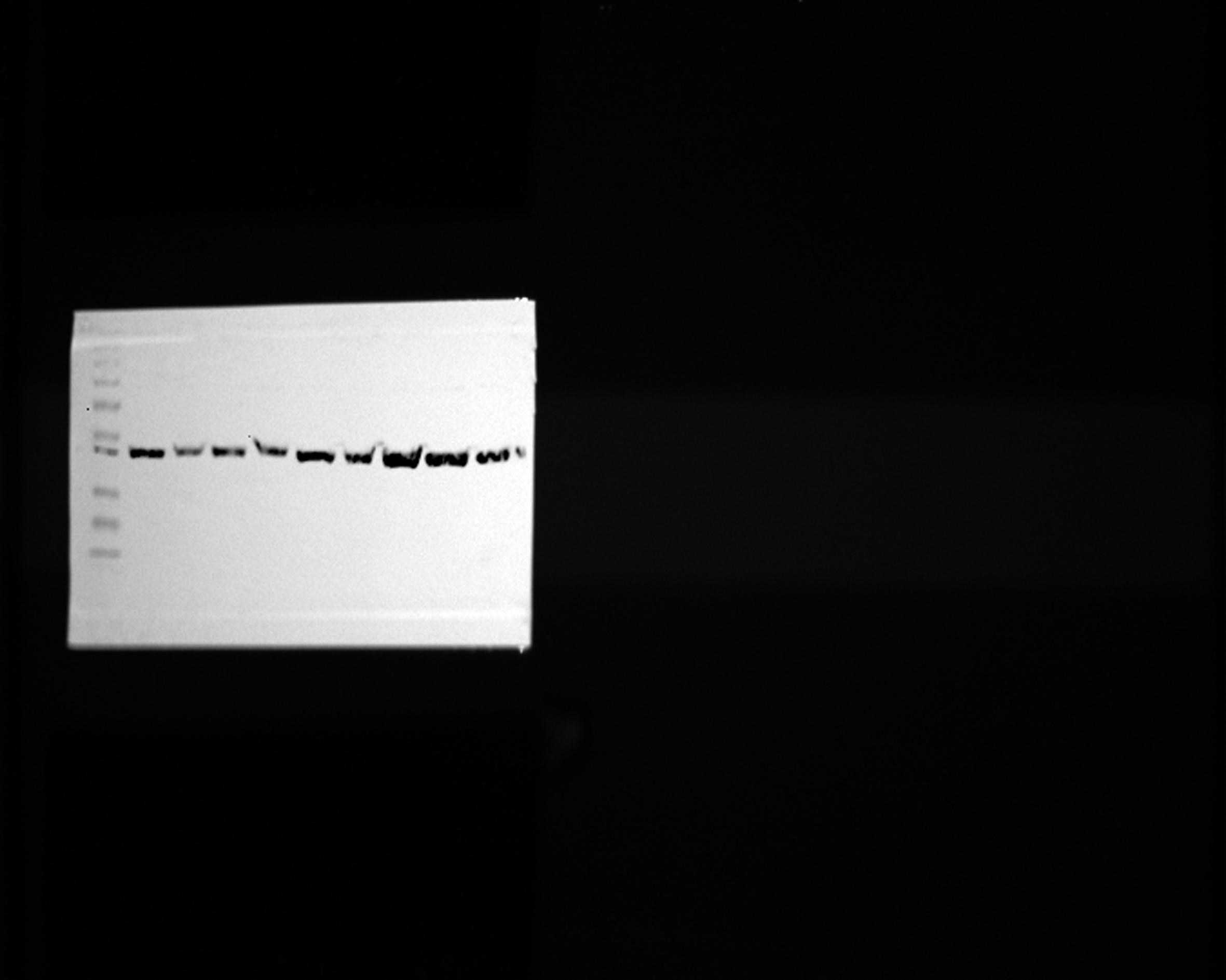

Supplement: Supplementary file 3 [file Image3.tif]

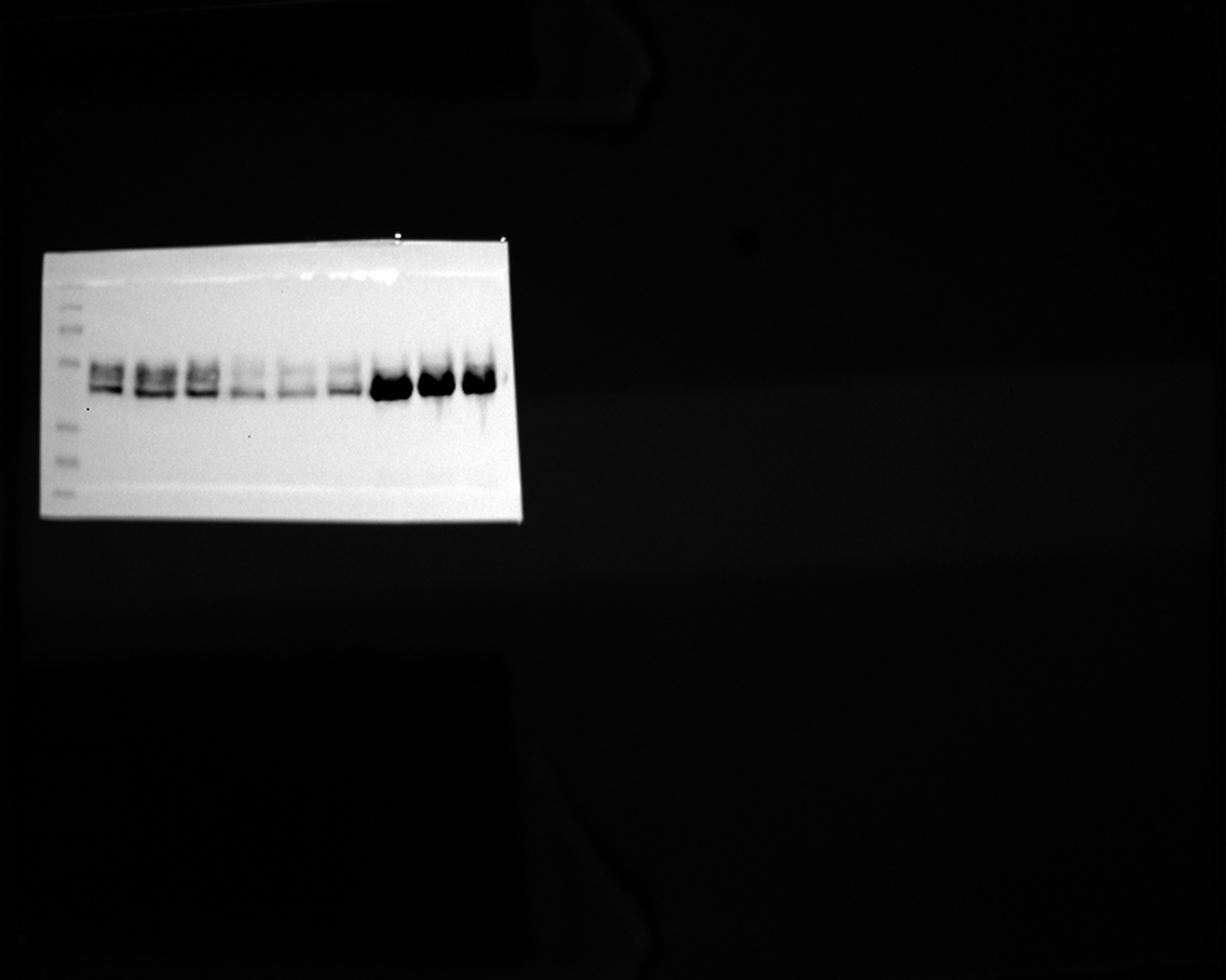

Supplement: Supplementary file 4 [file Image4.tif]

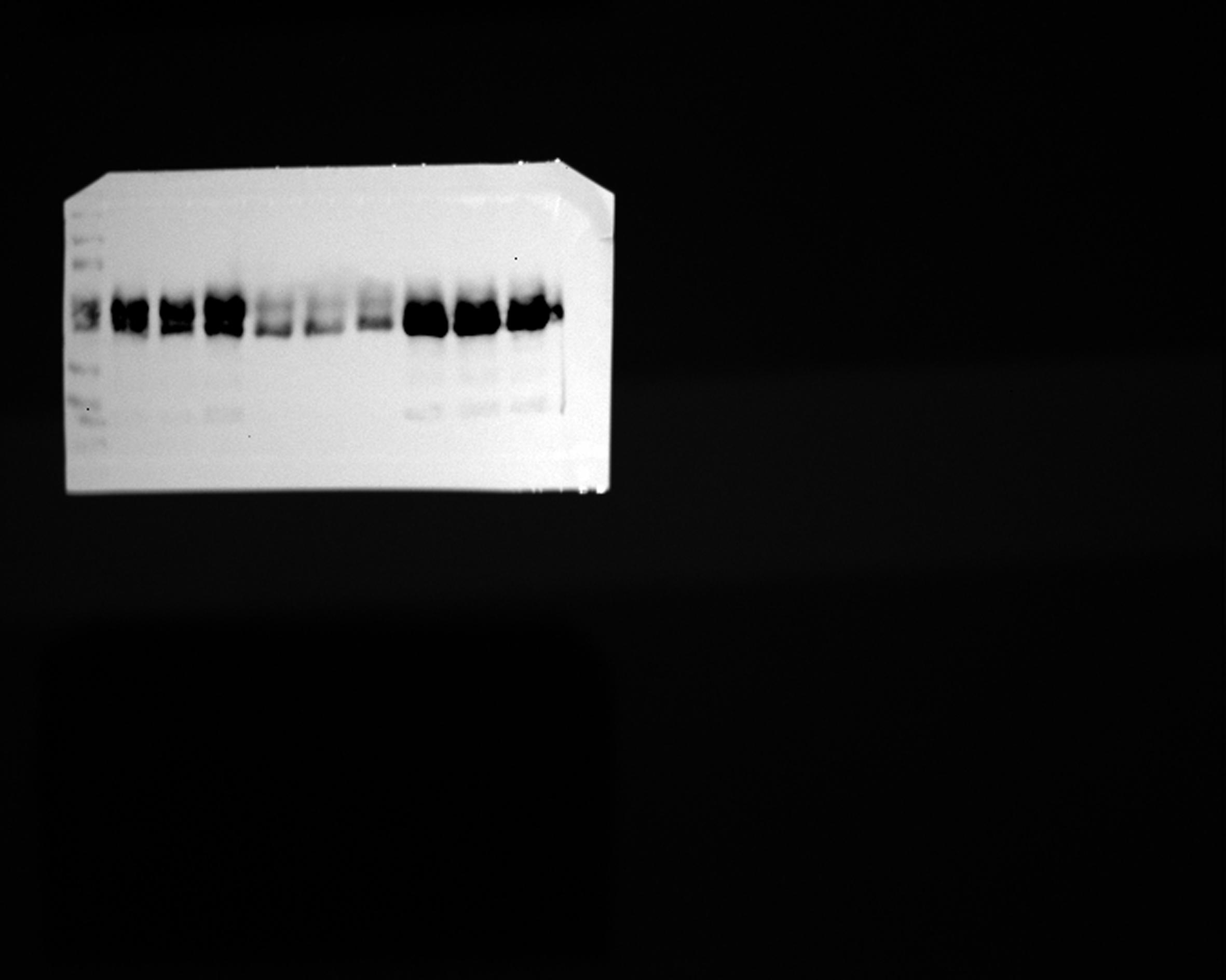

Supplement: Supplementary file 5 [file Image2.tif]

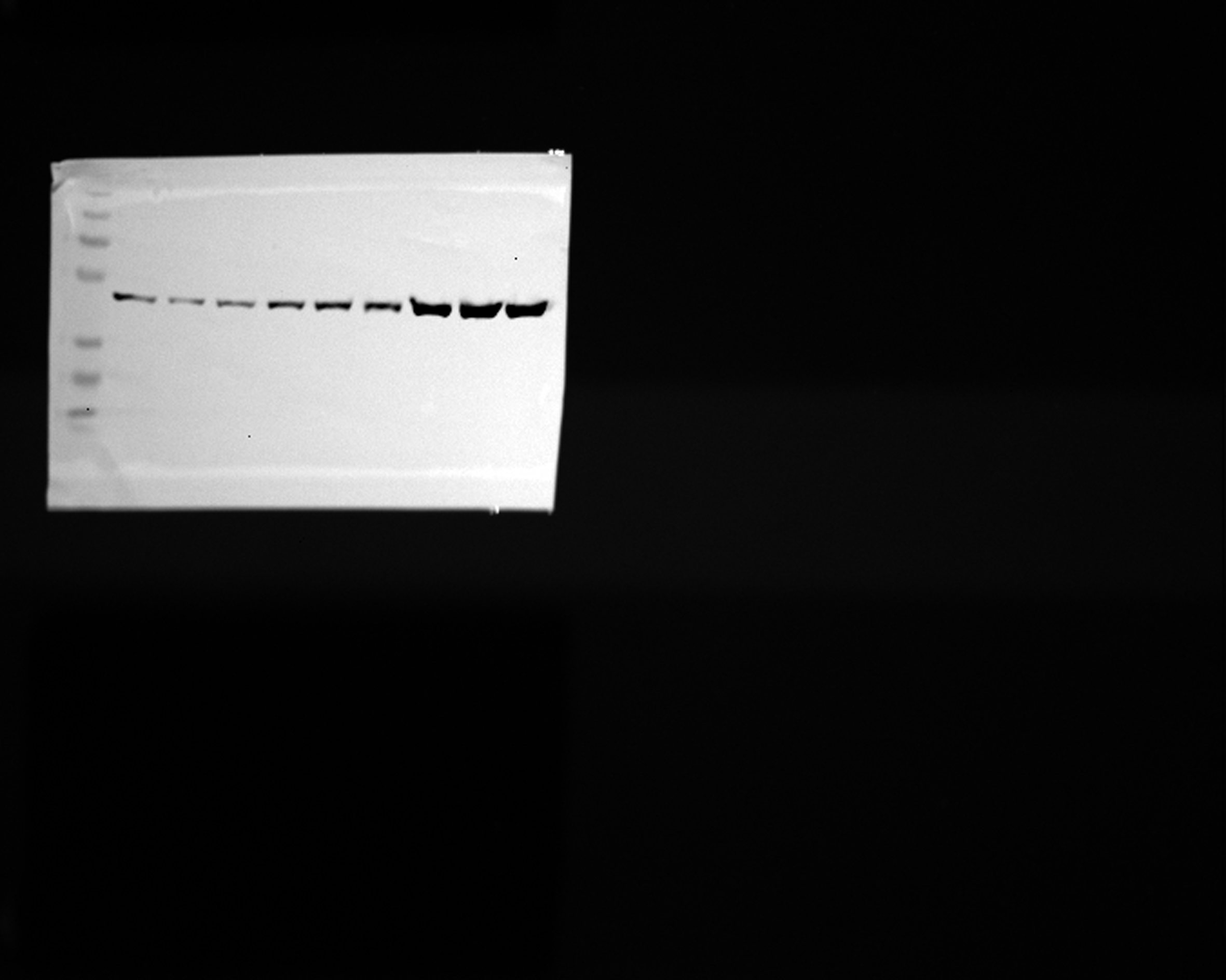

Supplement: Supplementary file 6 [file Image1.tif]

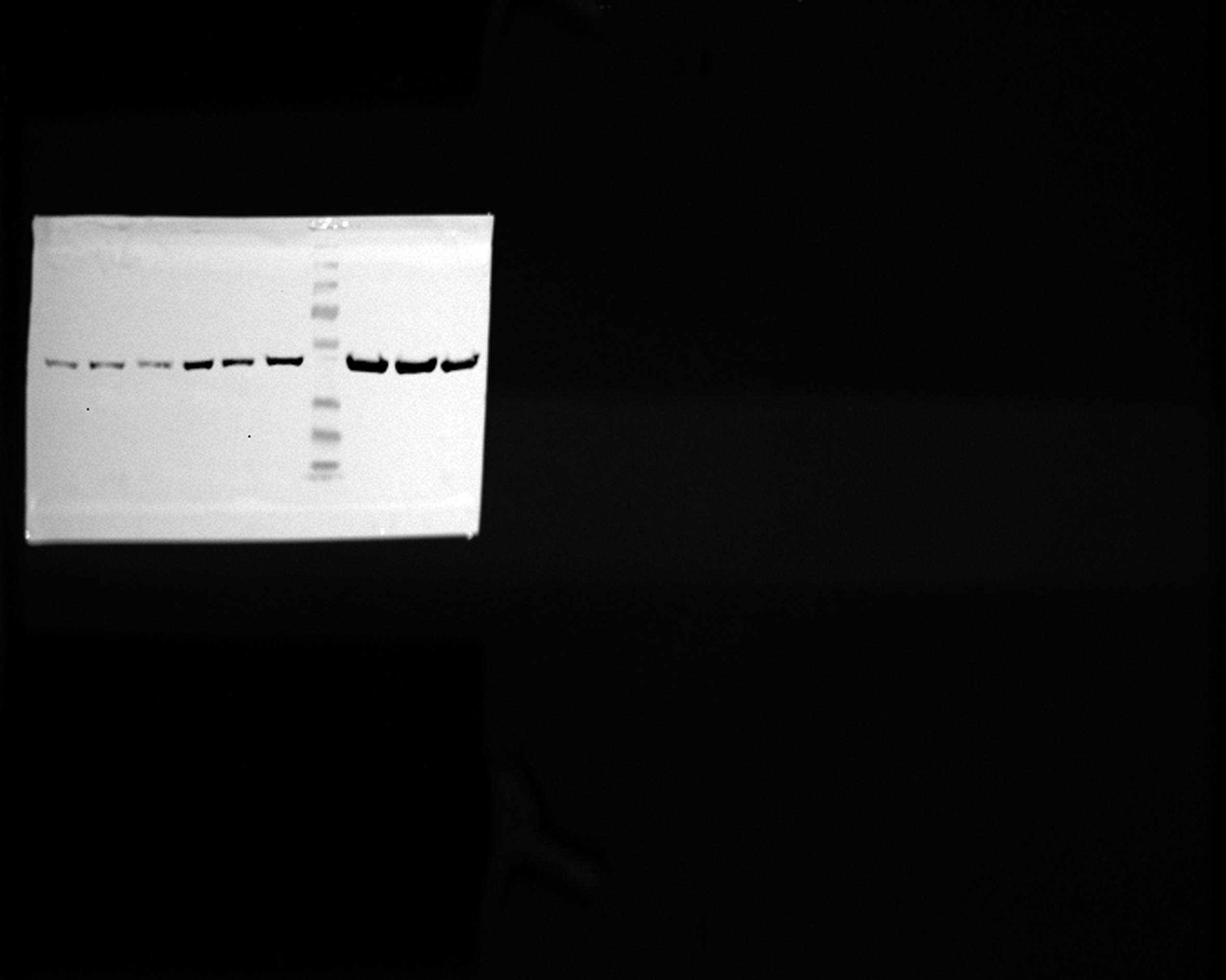

Supplement: Supplementary file 7 [file Image7.tif]

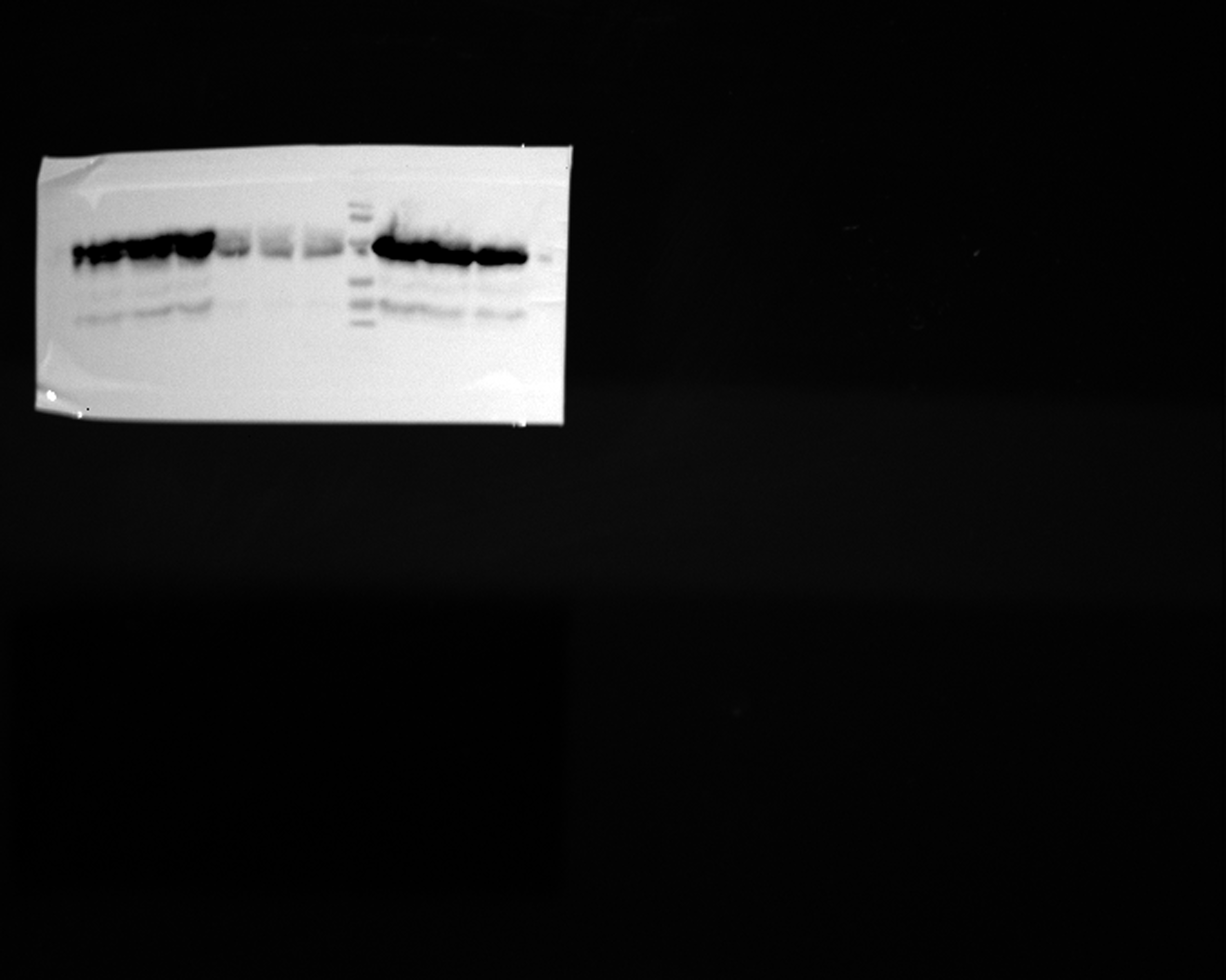

Supplement: Supplementary file 8 [file Image8.tif]

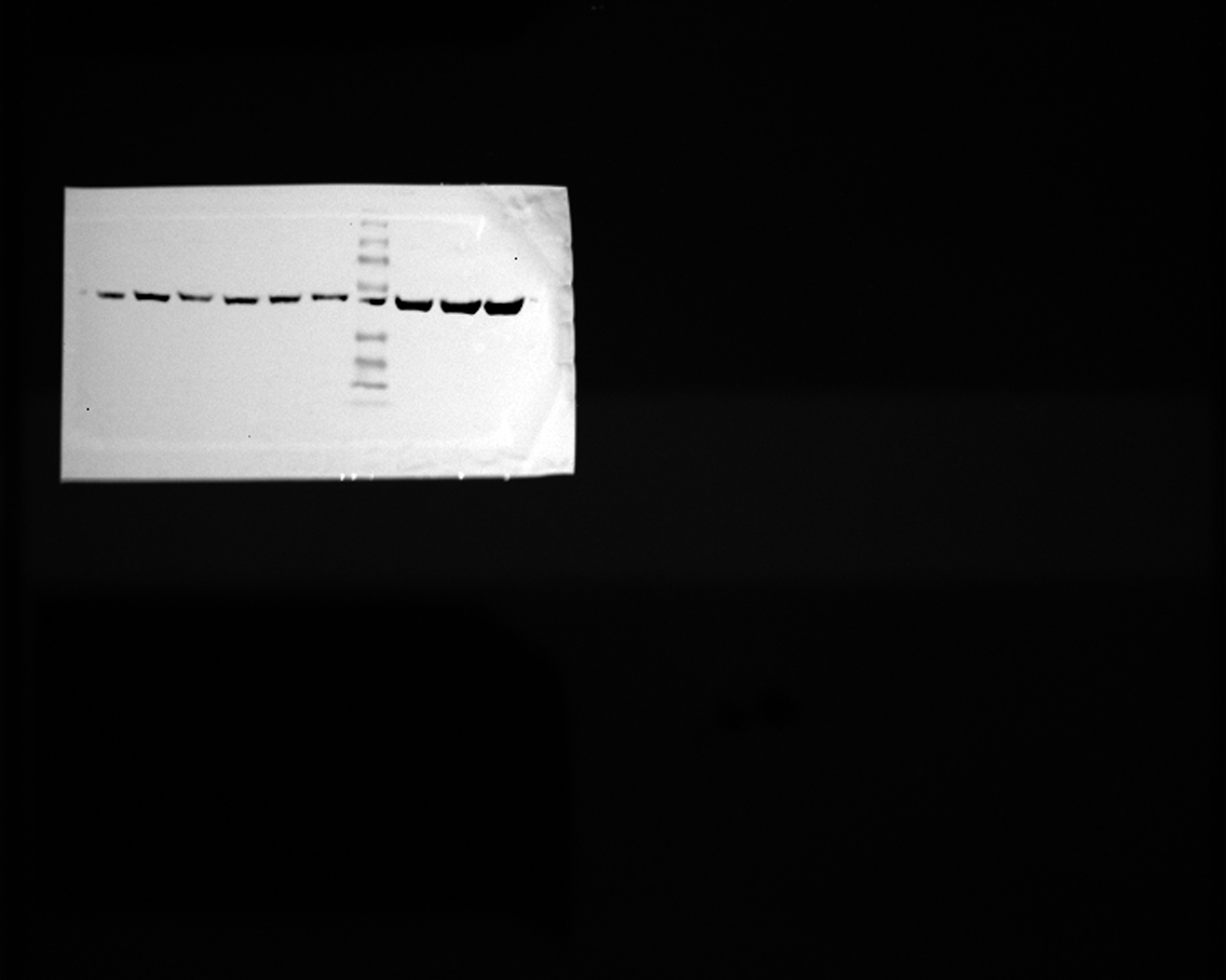

Supplement: Supplementary file 9 [file Image5.tif]
