# Supplementary material for: Network-level reprogramming of cell death pathways in colorectal cancer cells by combined thymoquinone and 5-fluorouracil treatment
Source: Front Mol Biosci. 2026 Jun 24;13:1864680. doi: 10.3389/fmolb.2026.1864680 (PMC13341502; doi:10.3389/fmolb.2026.1864680)
Supplement: Supplementary file 2 [file Supplementaryfile1.docx]

Supplementary Material

# Supplementary Figures and Tables


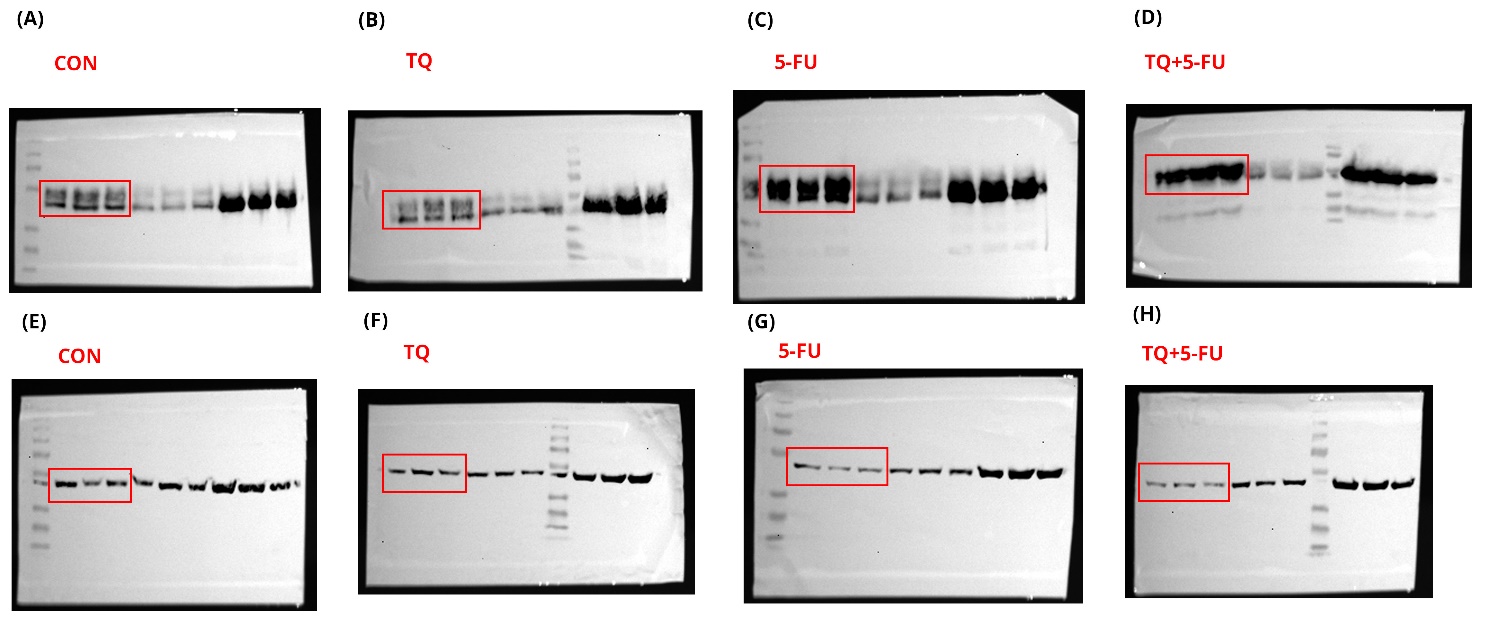


**Supplementary Figure S1.** Full-length, uncropped Western blot images corresponding to the data presented in Figure 14 of the main manuscript. Panels (A)–(D) show the original raw blot images for Fas. Panels (E)–(H) show the corresponding β-actin loading controls. Red boxes indicate the regions presented in the main figure. Unmarked lanes were not included in the analysis. Non-adjacent lanes are indicated in the main figure. CON – control (untreated cells); TQ – thymoquinone; 5-FU – 5-fluorouracil; TQ+5-FU – combined treatment.

**Supplementary Table S1.** Summary of previously published biological responses of RKO cells under the treatment conditions used in the present study.

| Compound | Concentration used in this study | Previously reported response in RKO cells | |
| --- | --- | --- | --- |
| TQ | **20 µM** | **Cell viability** | 76 ± 14% compared with untreated control;  Estimated IC₅₀ ≈ 37 μM |
|  |  | **DNA fragmentation** | 2.3 ± 0.22-fold increase relative to untreated controls |
|  |  | **Caspase activity** | No significant changes compared to control |
|  |  | **Flow cytometric analysis** | 14 ± 4% necrotic cells, 0.8 ± 0.5% apoptotic cells |
|  |  | **Gene expression** | No significant changes compared to control |
| 5-FU | **10 µg/mL (76.8 µM)** | **Cell viability** | 62 ± 21% compared with untreated control |
|  |  | **DNA fragmentation** | 1.74 ± 0.59-fold increase relative to untreated controls |
|  |  | **Caspase activity** | Caspase-3/7: 256 ± 13% of control levels  Caspase-8: 125 ± 1% of control levels |
|  |  | **Flow cytometric analysis** | No significant changes compared to control |
|  |  | **Gene expression** | *↑CASP3, ↑BAX, ↑FAS, ↑RIPK1* |
| TQ+5-FU | **20 µM  + 10 µg/mL** | **Cell viability** | 56 ± 14% compared with untreated control |
|  |  | **DNA fragmentation** | 4.7 ± 0.74-fold increase relative to untreated controls |
|  |  | **Caspase activity** | Caspase 3/7: 201 ± 30% of control levels |
|  |  | **Flow cytometric analysis** | 26 ± 3% necrotic cells, 2 ± 1% apoptotic cells |
|  |  | **Gene expression** | *↑CASP3, ↑CASP7, ↑BAX, ↑FAS, ↑RIPK1* |

Data derived from the previously published study by Kurowska et al. (2026). TQ – thymoquinone; 5-FU – 5-fluorouracil; TQ+5-FU – combined treatment, ↑ expression increase.

**Supplementary Table S2.** Shared differentially expressed genes and fold-change (FC) values in RKO colorectal cancer cells following thymoquinone (TQ), 5-fluorouracil (5-FU), or combined treatment (TQ+5-FU).

| Gene Symbol | Gene Name | FC (TQ) | FC (5-FU) | FC (TQ+5-FU) | Regulation Pattern |
| --- | --- | --- | --- | --- | --- |
| *ID2* | Inhibitor of DNA binding 2 | 6.732 | 3.939 | 12.760 | ↑ |
| *TNC* | Tenascin C | 2.519 | 2.218 | 2.570 | ↓ |
| *BIRC2* | Baculoviral IAP repeat containing 2 | 2.515 | 2.850 | 2.578 | ↑ |
| *SNX2* | Sorting nexin 2 | 2.336 | 3.102 | 2.295 | ↑ |
| *RIOK3* | RIO kinase 3 | 2.418 | 4.163 | 3.417 | ↑ |
| *IFRD1* | Interferon related developmental regulator 1 | 2.270 | 3.243 | 3.605 | ↑ |
| *RGS2* | Regulator of G protein signaling 2 | 4.464 | 2.082 | 4.034 | ↑ |
| *EPS8* | EGFR pathway substrate 8 | 2.052 | 2.611 | 2.258 | ↑ |
| *SP100* | SP100 nuclear antigen | 2.058 | 2.418 | 2.303 | ↑ |
| *ATP6V1C1* | ATPase H+ transporting V1 subunit C1 | 2.407 | 2.606 | 2.260 | ↑ |
| *TRIP4* | Thyroid hormone receptor interactor 4 | 2.014 | 2.203 | 2.295 | ↑ |
| *DNAJB4* | DnaJ heat shock protein family (Hsp40) member B4 | 2.510 | 2.744 | 2.572 | ↑ |
| *WDR47* | WD repeat domain 47 | 2.427 | 2.276 | 2.418 | ↑ |
| *FZD6* | Frizzled class receptor 6 | 2.249 | 2.241 | 2.182 | ↑ |
| *STK3* | Serine/threonine kinase 3 | 2.075 | 2.535 | 2.348 | ↑ |
| *ENPP4* | Ectonucleotide pyrophosphatase/phosphodiesterase 4 | 2.476 | 2.270 | 2.158 | ↑ |
| *RCBTB2* | RCC1 and BTB domain containing protein 2 | 2.301 | 4.101 | 3.319 | ↑ |
| *MAP3K8* | Mitogen-activated protein kinase kinase kinase 8 | 3.595 | 2.213 | 3.688 | ↑ |
| *EGR2* | Early growth response 2 | 2.061 | 3.302 | 4.187 | ↑ |
| *SERPINI1* | Serpin family I member 1 | 3.800 | 2.938 | 4.573 | ↑ |
| *HSPA4L* | Heat shock protein family A (Hsp70) member 4 like | 2.194 | 2.659 | 2.125 | ↑ |
| *HDAC9* | Histone deacetylase 9 | 2.027 | 3.179 | 2.817 | ↑ |
| *TDO2* | Tryptophan 2,3-dioxygenase | 3.096 | 3.029 | 3.145 | ↑ |
| *ZNF222* | Zinc finger protein 222 | 2.223 | 2.183 | 2.737 | ↑ |
| *ZNF165* | Zinc finger protein 165 | 2.540 | 2.910 | 3.192 | ↑ |
| *SERINC1* | Serine incorporator 1 | 2.223 | 2.114 | 2.262 | ↑ |
| *EIF5* | Eukaryotic translation initiation factor 5 | 2.731 | 2.144 | 2.356 | ↑ |
| *CLDND1* | Claudin domain containing 1 | 2.124 | 2.973 | 2.230 | ↑ |
| *RSRP1* | Arginine and serine rich protein 1 | 2.394 | 2.914 | 3.605 | ↑ |
| *FOS* | Fos proto-oncogene, AP-1 transcription factor subunit | 3.852 | 3.071 | 7.016 | ↑ |
| *HSDL2* | Hydroxysteroid dehydrogenase like 2 | 2.054 | 2.527 | 2.186 | ↑ |
| *ARC* | Activity regulated cytoskeleton associated protein | 4.090 | 2.615 | 8.537 | ↑ |
| *RGS20* | Regulator of G protein signaling 20 | 4.751 | 5.076 | 7.098 | ↑ |
| *CLK4* | CDC like kinase 4 | 2.435 | 2.442 | 2.114 | ↑ |
| *BIRC3* | Baculoviral IAP repeat containing 3 | 3.784 | 14.065 | 4.960 | ↑ |
| *CCNE2* | Cyclin E2 | 2.871 | 4.468 | 3.053 | ↑ |
| *RBM34* | RNA Binding Motif Protein 34 | 2.432 | 2.342 | 2.130 | ↑ |
| *BBS4* | Bardet-Biedl syndrome 4 | 2.061 | 2.489 | 2.447 | ↑ |
| *ATP10D* | ATPase phospholipid transporting 10D | 2.395 | 2.322 | 2.534 | ↑ |
| *ABCA5* | ATP binding cassette subfamily A member 5 | 2.101 | 2.882 | 2.012 | ↑ |
| *MYBL1* | MYB proto-oncogene like 1 | 3.209 | 6.072 | 4.697 | ↑ |
| *MIR22HG* | MIR22 host gene | 2.150 | 3.716 | 3.890 | ↑ |
| *ZNF468* | Zinc finger protein 468 | 2.483 | 2.957 | 2.472 | ↑ |
| *FAS* | Fas cell surface death receptor | 2.754 | 12.724 | 7.943 | ↑ |
| *YPEL5* | Yippee like 5 | 2.090 | 4.000 | 3.053 | ↑ |
| *TMEM2* | Transmembrane Protein 2 | 2.435 | 2.959 | 2.730 | ↑ |
| *EIF2AK3* | Eukaryotic translation initiation factor 2 alpha kinase 3 | 2.140 | 2.104 | 2.079 | ↑ |
| *ERAP2* | Endoplasmic reticulum aminopeptidase 2 | 2.956 | 3.965 | 2.124 | ↑ |
| *MCOLN3* | Mucolipin TRP cation channel 3 | 2.217 | 2.825 | 2.611 | ↑ |
| *C21orf91* | Chromosome 21 open reading frame 91 | 2.022 | 2.792 | 2.777 | ↑ |
| *SCYL2* | SCY1 like pseudokinase 2 | 2.663 | 2.344 | 2.370 | ↑ |
| *TAF9B* | TATA-box binding protein associated factor 9b | 2.809 | 2.332 | 2.044 | ↑ |
| *CYLD* | CYLD lysine 63 deubiquitinase | 2.469 | 4.905 | 3.725 | ↑ |
| *GSAP* | Gamma-secretase activating protein | 2.781 | 2.080 | 2.748 | ↑ |

↑ upregulated in all treatment groups; ↓ downregulated in all treatment groups.
